# Domain Mapping of Chondroitin/Dermatan Sulfate Glycosaminoglycans Enables Structural Characterization of Proteoglycans

**DOI:** 10.1016/j.mcpro.2021.100074

**Published:** 2021-03-20

**Authors:** Andrea Persson, Mahnaz Nikpour, Egor Vorontsov, Jonas Nilsson, Göran Larson

**Affiliations:** 1 Department of Laboratory Medicine, Sahlgrenska Academy at the University of Gothenburg, Sweden; 2 Proteomics Core Facility, Sahlgrenska Academy at the University of Gothenburg, Sweden; 3 Laboratory of Clinical Chemistry, Sahlgrenska University Hospital, Västra Götaland Region, Sweden

**Keywords:** mass spectrometry (MS), LC-MS/MS, higher-energy collision dissociation (HCD), chondroitin/dermatan sulfate (CS/DS), glycan, glycomics, CgA, chromogranin-A, CS/DS, chondroitin/dermatan sulfate, DBA, dibutylamine, dp, degree of polymerization, GAG, glycosaminoglycan, Gal, galactose, GalNAc, *N*-acetylgalactosamine, GlcA, glucuronic acid, HA, hyaluronic acid, HCD, higher-energy collision dissociation, HexA, hexuronic acid, HS, heparan sulfate, IAPP, islet amyloid polypeptide, IdoA, iduronic acid, NCE, normalized collision energy, Neu5Ac, *N*-acetylneuraminic acid, NRE, nonreducing end, PG, proteoglycan, TIC, total ion chromatogram, XIC, extracted ion chromatogram, Xyl, xylose

## Abstract

Of all posttranslational modifications known, glycosaminoglycans (GAGs) remain one of the most challenging to study, and despite the recent years of advancement in MS technologies and bioinformatics, detailed knowledge about the complete structures of GAGs as part of proteoglycans (PGs) is limited. To address this issue, we have developed a protocol to study PG-derived GAGs. Chondroitin/dermatan sulfate conjugates from the rat insulinoma cell line, INS-1832/13, known to produce primarily the PG chromogranin-A, were enriched by anion-exchange chromatography after pronase digestion. Following benzonase and hyaluronidase digestions, included in the sample preparation due to the apparent interference from oligonucleotides and hyaluronic acid in the analysis, the GAGs were orthogonally depolymerized and analyzed using nano-flow reversed-phase LC-MS/MS in negative mode. To facilitate the data interpretation, we applied an automated LC-MS peak detection and intensity measurement *via* the Proteome Discoverer software. This approach effectively provided a detailed structural description of the nonreducing end, internal, and linkage region domains of the CS/DS of chromogranin-A. The copolymeric CS/DS GAGs constituted primarily consecutive glucuronic-acid-containing disaccharide units, or CS motifs, of which the *N*-acetylgalactosamine residues were 4-*O*-sulfated, interspersed by single iduronic-acid-containing disaccharide units. Our data suggest a certain heterogeneity of the GAGs due to the identification of not only CS/DS GAGs but also of GAGs entirely of CS character. The presented protocol allows for the detailed characterization of PG-derived GAGs, which may greatly increase the knowledge about GAG structures in general and eventually lead to better understanding of how GAG structures are related to biological functions.

## Highlights

• Protocol developed to structurally characterize glycosaminoglycans of proteoglycans.
• Comprehensive characterization of cellular glycosaminoglycan structures.
• Relative quantification of nonreducing end, internal, and linkage region domains.
• Overall chondroitin/dermatan sulfate glycosaminoglycan structures of chromogranin-A.

## Graphical Abstract

**Figure:**
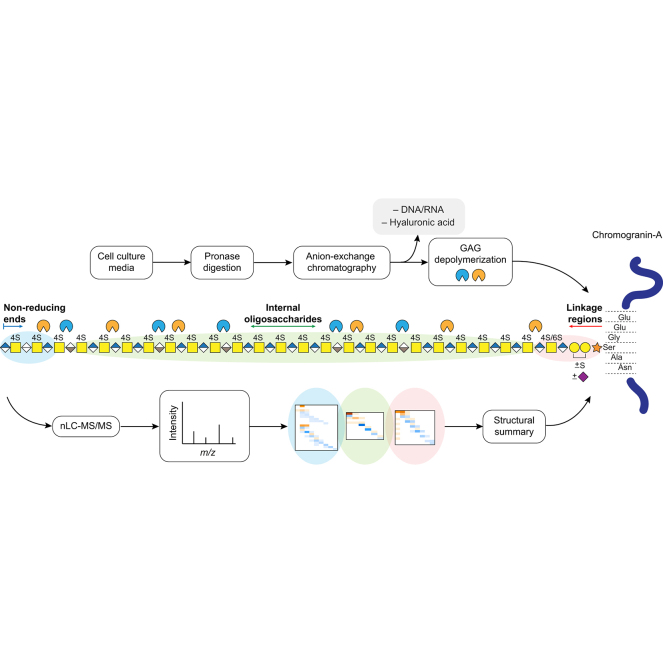

## In Brief

Glycosaminoglycans (GAGs) remain one of the most challenging posttranslational modifications to study, much due to their structural complexity and heterogeneity, and new methods for analysis are therefore required. We have developed a protocol for enrichment and structural characterization of GAGs of proteoglycans using nLC-MS/MS. We provide detailed information on the nonreducing end, internal, and linkage region GAG domains and use the data to determine an overall GAG structure of chromogranin-A of rat INS-1832/13 cells.

Proteoglycans (PGs) are glycoconjugates where the glycan part comprises highly complex polysaccharides known as glycosaminoglycans (GAGs). PGs are produced by most cells and involved in a variety of biological processes (1, 2), which make them important to study from both structural and functional perspectives. However, due to the heterogeneity of the GAGs and methodological limitations, the detailed structures of PG-derived GAGs from cells and tissues remain largely unknown (3, 4).

**Fig. 1:**
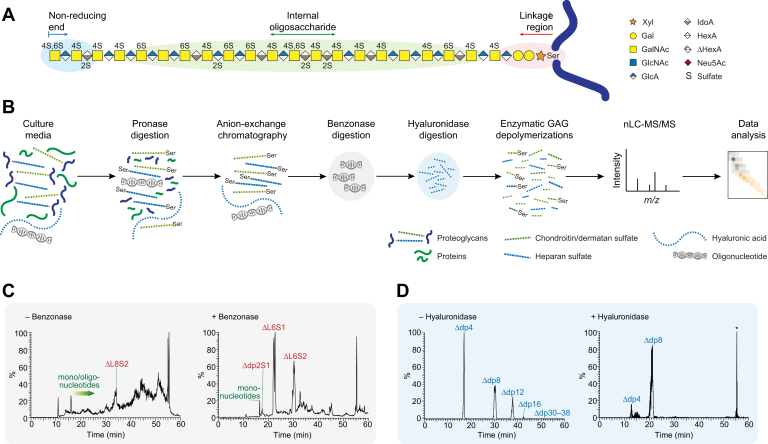
**General CS/DS structure and our employed workflow for structural domain mapping of PG-derived GAGs.***A*, structure and domain distribution of a typical PG-derived CS/DS GAG. *B*, schematic view of GAGDoMa sample preparation and analysis. *C* and *D*, rationale for including benzonase and hyaluronidase digestions in the sample preparation. Total ion chromatograms before and after benzonase digestion (*C*) display interference of oligonucleotides and indicate incomplete GAG depolymerization with chondroitinase ABC without benzonase digestion. Extracted ion chromatograms (XICs) at *m/z* 757.22 before and after hyaluronidase digestion (*D*) display a reduction in HA variants upon digestion. The peak indicated by an asterisk arises from background noise.

Chondroitin/dermatan sulfate (CS/DS) corresponds to one class of GAGs (Fig. 1*A*) commonly constituting 25–100 repeating disaccharide units (degree of polymerization, dp50–200), where the CS motifs comprise glucuronic acid (GlcA) and *N*-acetylgalactosamine (GalNAc) units (-4GlcAβ3GalNAcβ-), and DS motifs comprise iduronic acid (IdoA) and GalNAc units (-4IdoAα3GalNAcβ-) as a result of enzymatic epimerization of GlcA to IdoA (5, 6). Further modification of the carbohydrate backbone involves 2-*O*-sulfation of GlcA and IdoA residues and 4-*O*- and 6-*O*-sulfation of GalNAc residues. The tetrasaccharide linkage region, -4GlcAβ3Galβ3Galβ4Xylβ-*O*-, found closest to the PG protein core (Fig. 1*A*), can be modified by *O*-sulfation of both Gal residues, sialylation of the first Gal residue from the reducing end, and phosphorylation of Xyl (7, 8). Altogether, this results in an enormous structural diversity of the CS/DS GAGs.

To obtain a structural overview of the GAGs, the GAGs are routinely enzymatically depolymerized to disaccharides and analyzed by HPLC or LC-MS (9, 10). Alternatively, glycoproteomics approaches may be used to provide detailed information about the linkage regions (7, 8, 11), but not the internal and nonreducing end (NRE) GAG domains. For the MS analysis of oligosaccharides and the intact GAGs of bikunin and decorin (12, 13), extensive purification has been required to reduce sample heterogeneity and obtain a limited number of similarly structured GAGs. Slightly different strategies are required to investigate biological samples with complex mixtures of PG-derived GAGs, aiming at the analysis of all three GAG domains: NREs, internal oligosaccharides, and linkage regions. We recently reported the *G*lycos*a*mino*g*lycan *Do*main *Ma*pping approach, GAGDoMa, based on orthogonal depolymerization of GAGs into the three domains, followed by reversed-phase dibutylamine (DBA) ion-paring chromatography with negative mode higher-energy collision dissociation (HCD) tandem mass spectrometry (MS/MS) for structural characterization of the domains (14). This approach enabled detailed characterization, identification, and relative quantification of a wide range of GAG structures. CS/DS primed on xylosides, chemical compounds that induce and amplify the GAG production by cells, was used as the model for developing the approach, but the long-term goal was to be able to characterize also PG-derived GAGs in a corresponding manner.

Here, we further developed the GAGDoMa approach for structural characterization of PG-derived GAGs using the media from cultured cells (Fig. 1*B*). Rat INS-1832/13 cells were selected since we have studied them in detail and found that they produce mainly one PG, chromogranin-A (CgA; Uniprot P10354), which appears to have only one GAG site, Ser-433, and is readily secreted into the culture medium (M.N., unpublished results). In addition, CgA is an important PG that serves as a key player in dense-core granule biogenesis in endocrine and neuroendocrine cells (15), and CgA and CgA-derived polypeptides appear as biomarkers for several pathological conditions including diabetes and its complications, different types of cancer, and cardiovascular diseases (16, 17). We show here that GAGDoMa offers detailed characterization and relative quantification of all three GAG domains: the NREs, internal oligosaccharides, and linkage region oligosaccharides. In addition, the secretory pattern of the INS-1832/13 cells allowed us to conclude an overall CS/DS GAG structure of CgA.

## Experimental Procedures

### Cell Culture

Rat insulinoma cells, INS-1832/13, provided by prof. Lena Eliasson (Lund University, Sweden), were cultured as monolayers in RPMI 1640 medium supplemented with 2 mM L-glutamine, 1 mM sodium pyruvate, 10% FBS, 10 mM HEPES, 100 units/ml penicillin, 100 μg/ml streptomycin, and 50 μM 2-mercaptoethanol at 37 °C in 5% CO_2_ in air atmosphere. The cells were routinely verified as *mycoplasma* free.

### Preparation of Glycomics Samples

Confluent INS-1832/13 cells were washed three times with PBS and incubated with fresh culture medium without FBS for 16 h. The culture media of 4–5 x 10^7^ cells were used for the sample preparations. The culture media were collected and centrifuged free from cells and debris, freeze-dried and dissolved in ddH_2_O, desalted using PD-10 (GE Healthcare), and freeze-dried again. The remaining pellet was dissolved in 10 mM Tris-HCl, pH 8.0, 1 mM CaCl_2_, 2% SDS, and 2 mg pronase (EC 3.4.24.4; Sigma-Aldrich) was added. After 16 h of incubation at 55 °C, residual enzyme activity was inactivated at 100 °C for 10 min. GAGs were then enriched using strong anion-exchange chromatography as previously described (11), except from the elution step that was performed in one step using 1.6 M NaCl, 50 mM Tris-HCl, pH 8.0. Using a 10 kDa molecular weight cutoff Slide-a-lyzer cassette (Thermo Scientific), the eluate was dialyzed to ddH_2_O in two steps (0.15 M NaCl for 2 h, followed by ddH_2_O for 16 h at 4 °C). As determined by the 1,9-dimethyl-methylene blue method (18), 2 μg of GAGs was depolymerized using chondroitinase ABC, chondroitinases AC-I and -II (AC), or chondroitinase B, or heparinases II and III for 16 h as previously described (19). In addition, 20 μg of GAGs was subjected to benzonase (EC 3.1.30.2) digestion (20 mU; Sigma-Aldrich) in 50 mM Tris-HCl, pH 8.0, 1 mM CaCl_2_, 2 mM MgCl_2_ for 2 h at 37 °C, and then heat inactivation and centrifugation at 500 x g for 2 min. The supernatant was desalted using 10 kDa molecular weight cutoff Spin-X UF concentrator (Corning) by washing thrice with ddH_2_O (each centrifugation 5000 x g, 5 min), and the GAGs were depolymerized. Another 20 μg of GAGs was subjected to benzonase digestion as described above, followed by hyaluronic acid (HA) digestion by stepwise addition of 300 mU hyaluronidase from *Streptomyces hyalurolyticus* (EC 4.2.2.1; Sigma-Aldrich) every hour for 4 h and then a final incubation for 16 h at 37 °C, heat inactivation, and desalting using Spin-X UF concentrator. Finally, the GAG samples were depolymerized using the abovementioned lyases. HA, 4-*O*-sulfated CS (Sigma-Aldrich), and defructosylated K4 polysaccharide, or chondroitin, (provided by Dr Emil Tykesson, Lund University, Sweden) were used as controls for enzymatic activity.

### nLC-MS/MS

In total, 0.15–2 μg of depolymerized GAGs was used for each analytical run. The nLC-MS/MS setup has previously been described in detail (14). Briefly, GAGs were trapped on a 2 cm × 100 μm Acclaim PepMap C18 precolumn, separated on a 30 cm × 75 μm C4 column or C18 column using a stepwise elution gradient from 0% to 70% methanol with 5 mM DBA and 8 mM acetic acid at 300 nl/min over 60 min, and analyzed on an LTQ Orbitrap Elite mass spectrometer (Thermo Fisher Scientific) operated in the negative electrospray ionization mode. For the MS^1^ only analysis, the spectra were acquired in the *m/z* range 220–2000 at 120,000 resolution, and for the MS^2^ analysis, precursor ions were scanned in the *m/z* range 220–2000 at 60,000 resolution in MS^1^, followed by the HCD-MS^2^ spectra of the five most abundant precursor ions, each with normalized collision energies (NCEs) at 60%, 70%, and 80%. The MS^2^ spectra were acquired in centroid mode in the *m/z* range 100–2000 at 15,000 resolution. Dynamic exclusion was disabled and precursor ions with unassigned charge states were rejected.

### Data Analysis

The nLC-MS/MS data were processed using the Xcalibur software (Thermo Fisher Scientific). The MS^2^ data were analyzed manually, and the results were used to generate lists containing the theoretical monoisotopic masses of possible GAG products. LC-MS features were detected and quantified using the Minora Feature Detection node within Proteome Discoverer version 2.4 (Thermo Fisher Scientific) and exported as tables containing data on the monoisotopic precursor ion *m/z* with charge state, chromatographic retention time, and precursor ion intensity for each LC-MS feature. Using an in-house Python script and a mass error tolerance of 15 ppm, the monoisotopic masses of the LC-MS features were matched against the theoretical monoisotopic masses within the assembled lists. The intensities of coeluting precursor ions that had the same glycan composition but differed by the charge states, number of DBA adducts, and sulfate losses were added together. All hits were verified manually by retention times and MS^2^ characteristics. Typical chromatograms and spectra were selected for the data presentation. Precursor ion masses were given as the monoisotopic masses to four decimal places, and fragment ion masses as the highest intensity isotope peak to two decimal places. Glycan symbols were depicted according to the Symbol Nomenclature for Glycans (20).

### Experimental Design and Statistical Rationale

For relative quantification, each sample was analyzed as three technical replicates. Statistics were performed and graphs generated using GraphPad Prism version 8.4.2 (GraphPad software). Heatmap cell data correspond to the mean relative intensity after each enzymatic depolymerization calculated as described in *Data analysis*.

## Results

### Method Development of GAGDoMa for PG-Derived GAGs

When developing a glycomics approach for domain mapping of PG-derived GAGs, not only the analytical method needs to be taken into consideration, but also the sample preparation. The goal was to keep the protocol as simple as possible to avoid losses of GAG material, but still gain representative samples pure enough for effective data interpretation. The initial protocol was similar to the one developed for PG glycopeptide preparations (11), which involved protein digestion, strong anion-exchange chromatography, and GAG depolymerization. However, instead of trypsin digestion, which is suitable for proteomics and glycoproteomics where peptide sequences of 5–30 amino acids are desirable (21), extensive digestion with pronase, a nonspecific protease, was performed to more completely hydrolyze the proteins to reduce the number of peptide variants, increase the analytical sensitivity, and facilitate the data interpretation of the linkage region products. Enzymatic digestion of the protein was preferred to chemical removal of the protein part, such as β-elimination, which can cause peeling of the glycan especially since relatively small amounts of biological starting material were used (12, 13). GAG depolymerization using bacterial lyases cleaves the GAGs into terminal NREs, internal oligosaccharides, and linkage regions comprising the linkage region tetrasaccharide (Fig. 1*A*). Due to the eliminative mechanism of the lyases, the cleavage between GalNAc and HexA residues generates 4,5-unsaturated hexuronic acid (ΔHexA) residues. The terminal NRE saccharide is either a GalNAc or HexA residue, where the HexA residues are distinguishable from the ΔHexA residues of the internal oligosaccharides by a mass of –18.0106 u. Depending on the specificity of the enzymes and the exact GAG structures, oligosaccharides of different lengths are expected; for example, chondroitinase ABC cleaves CS/DS at both GlcA and IdoA residues (22, 23), whereas chondroitinase AC and chondroitinase B cleave CS/DS at GlcA and IdoA residues, respectively.

Using our established glycoproteomics approach (7, 11, 24), we recently characterized the PGs produced by INS-1832/13 cells and showed that CgA was the dominating CSPG produced by the cells (M.N., unpublished results). From the glycopeptide analysis, we observed that the majority of the PGs were found in the culture media (M.N., unpublished results), thus only the culture media were used in the current study aiming to accomplish an in-depth structural analysis of the entire GAG structures of cellular PGs. Apart from CgA, only islet amyloid polypeptide (IAPP; Uniprot P12969) was observed as a secreted CSPG; however, IAPP appeared to a much lesser extent than CgA (∼94% CgA; ∼6% IAPP; based on relative intensities of the precursor ions corresponding to the non-, mono-, and disulfated primary glycopeptides of each PG; supplemental Fig. S1).

Endonucleases, such as benzonase, are commonly included in GAG sample preparations from fluids and tissues to remove nucleic acids (25, 26, 27, 28, 29). To investigate whether nucleic acid digestion had any impact on the GAG sample preparation from INS-1832/13 cells, the total ion chromatograms (TICs) of chondroitinase ABC-depolymerized CS/DS products before and after benzonase treatment were compared (Fig. 1*C*, supplemental Fig. S2, *A* and *B*). The INS-1832/13 cells appeared to produce a considerable number of identifiable oligonucleotides (supplemental Figs. S2 and S3), which were evidently obstructing the data interpretation. In addition, the presence of the oligonucleotides inhibited the GAG depolymerization as concluded from the predominance of octasaccharide linkage region variants (ΔL8) after chondroitinase ABC depolymerization instead of the expected hexasaccharide linkage region variants (ΔL6; Fig. 1*C* and supplemental Fig. S2*A*). The benzonase digestion efficiently reduced the number and size of the oligonucleotides and importantly promoted complete chondroitinase ABC depolymerization (Fig. 1*C* and supplemental Fig. S2*B*). Thus, benzonase digestion was included as an essential step in the PG-derived GAG sample preparations.

**Fig. 2:**
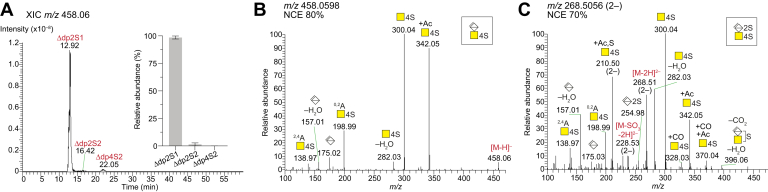
**Disaccharide composition of CS/DS from INS-1832/13 cells.***A*, XIC at *m/z* 458.06 corresponding to internal oligosaccharides carrying one sulfate group per disaccharide, Δdp2nSn (n = 1, 2, 3, …), displays the CS/DS di- and oligosaccharides generated after chondroitinase ABC depolymerization. The *inset* shows the relative abundance of the different CS/DS di- and tetrasaccharides as calculated using an automated search routine (mean ± SD). *B* and *C*, HCD-MS^2^ spectra of CS/DS disaccharides from INS-1832/13 cells after chondroitinase ABC depolymerization; the monosulfated disaccharide, Δdp2S1, displayed as the [M-H]^–^ precursor ion at *m/z* 458.0598 at normalized collision energy (NCE) 80% corresponding to ΔHexAGalNAc4S (*B*), and the disulfated disaccharide, Δdp2S2, displayed as the [M-2H]^2–^ precursor ion at *m/z* 268.5056 at NCE 70%, corresponding to ΔHexA2SGalNAc4S (*C*). Mass accuracies of precursor and fragment ions are found in supplemental Tables S1 and S2.

In addition to oligonucleotides, nonsulfated GAG-derived oligosaccharides were detected in the samples after chondroitinase ABC and AC depolymerizations, but not after chondroitinase B depolymerization (Figs. 1*D* and supplemental Fig. S4). HA, another class of GAGs comprising nonsulfated -4GlcAβ3GlcNAcβ-units, is known to be susceptible to depolymerization by chondroitinases ABC and AC-II (30, 31). Thus, to determine whether the nonsulfated oligosaccharides originated from HA or from incompletely depolymerized nonsulfated CS motifs, HA and defructosylated K4 polysaccharide (dK4, or chondroitin) were subjected to digestions using hyaluronidase and chondroitinase ABC, and the products were analyzed (supplemental Figs. S4–S6). The HA sample was susceptible to both the hyaluronidase and chondroitinase ABC digestions, whereas the chondroitin sample was susceptible only to the chondroitinase ABC digestion (supplemental Fig. S5). By comparing the MS^2^ spectra of HA- and chondroitin-derived oligosaccharides with the ones of the nonsulfated oligosaccharides from INS-1832/13 cells (supplemental Fig. S6), the nonsulfated oligosaccharides in the INS-1832/13 sample were concluded to be HA-derived (32, 33). Since both chondroitinases ABC and AC are reported to depolymerize HA, we suspected that the depolymerization of CS/DS may even be competitively inhibited by the presence of HA (34). Thus, an HA-removing step using hyaluronidase digestion was included in the GAG sample preparation, which distinctly reduced but not completely removed the HA (Fig. 1*D* and supplemental Fig. S7). By careful inspection of the MS data, we concluded that an adequate level of CS/DS depolymerization was acquired (Fig. 2*A*), and the HA digestion protocol was therefore not further optimized.

For the LC separation of oligosaccharides, we have previously been using only a C18 column. However, to investigate if we could resolve larger GAG oligosaccharides by reducing the retention times, a less hydrophobic C4 column was introduced, and indeed the separation and detection of longer GAG structures were enabled (supplemental Fig. S8). The remaining conditions of the previously reported MS/MS setup were maintained for the PG-derived GAG analysis (14).

To automate the quantification of the precursor ion signals in the LC-MS data, we utilized the Minora Feature Detector node of the Proteome Discoverer software (35). This choice was based on our previous in-house experience of using the node for the peptide quantification workflows in proteomic applications (36). The Minora Feature Detector node enabled the detection and quantification of LC-MS precursor ion features and yields, such as the monoisotopic *m/z*, charge, retention time, and maximal intensity for each chromatographic peak. When matching the assembled list of theoretical monoisotopic *m/z* values of possible structures (supplemental Table S4) with the experimental ones, the mass error tolerance was set to 15 ppm; however, the observed mass error was considerably lower for most ions. For example, the average mass error for all precursor ions in supplemental Table S1 is 0.9 ppm with a standard deviation of 1.2 ppm. To evaluate the automated quantification, the relative abundances of glycan structures from commercially available 4-*O*-sulfated CS depolymerized with chondroitinase ABC were compared with the corresponding manually obtained relative abundances, showing similar results (supplemental Fig. S9). Furthermore, the reproducibility between the runs was studied, and the level of variance observed was concluded acceptable (supplemental Fig. S9). All relative quantitative results presented henceforth are based on the automatically quantified data (raw data are found in supplemental Table S3).

### Disaccharide Composition

To obtain an overview of the CS/DS structures produced by INS-1832/13 cells, the internal oligosaccharides generated after chondroitinase ABC depolymerization were initially analyzed. Chondroitinase ABC cleaves CS/DS at both GlcA and IdoA residues, therefore, disaccharides (Δdp2) are expected as the only internal oligosaccharide product when complete depolymerization is achieved. By studying the extracted ion chromatogram (XIC) at *m/z* 458.06 (n–), which corresponds to internal oligosaccharides carrying one sulfate group per disaccharide, Δdp2nSn (n = 1, 2, 3, …), the majority of the internal oligosaccharides were identified as disaccharides (Fig. 2*A*). Only trace amounts (<1%) of tetrasaccharides at *m/z* 458.06 (2–), Δdp4S2, and no larger structures were detected, implying that close to complete depolymerization was achieved. The monosulfated disaccharides constituted the major product (∼98%) after chondroitinase ABC depolymerization, followed by the disulfated disaccharides, Δdp2S2 (∼1%), which appeared at *m/z* 458.06 due to in-source sulfate loss (∼60%). All precursor ions for each identified glycan, not only the ones at *m/z* 458.06, were taken into account in the relative abundance estimation using our automated search routine (inset, Fig. 2*A*).

We have previously demonstrated the fragmentation characteristics of all the CS/DS disaccharides using the GAGDoMa approach, despite the lack of baseline separation of the isomeric ones (14). The current MS^2^ spectra revealed that the monosulfated disaccharides in CS/DS from INS-1832/13 cells corresponded only to ΔHexAGalNAc4S, since no MS^2^ spectra displaying the fragmentation characteristics of the ΔHexAGalNAc6S disaccharide were detected (Fig. 2*B*). The disulfated disaccharides corresponded to ΔHexA2SGalNAc4S (Fig. 2*C*) (37, 38). Similarly to previous studies (7, 14), different NCEs were applied sequentially on the same precursor ion since the NCE and charge state have been shown to greatly impact the fragmentation pattern of the precursor ions. Thus, the NCEs were selected accordingly (14), and, in general, higher NCEs were applied for lower charge states, and lower NCEs for higher charge states.

### Nonreducing Ends and Internal Oligosaccharides

**Fig. 3:**
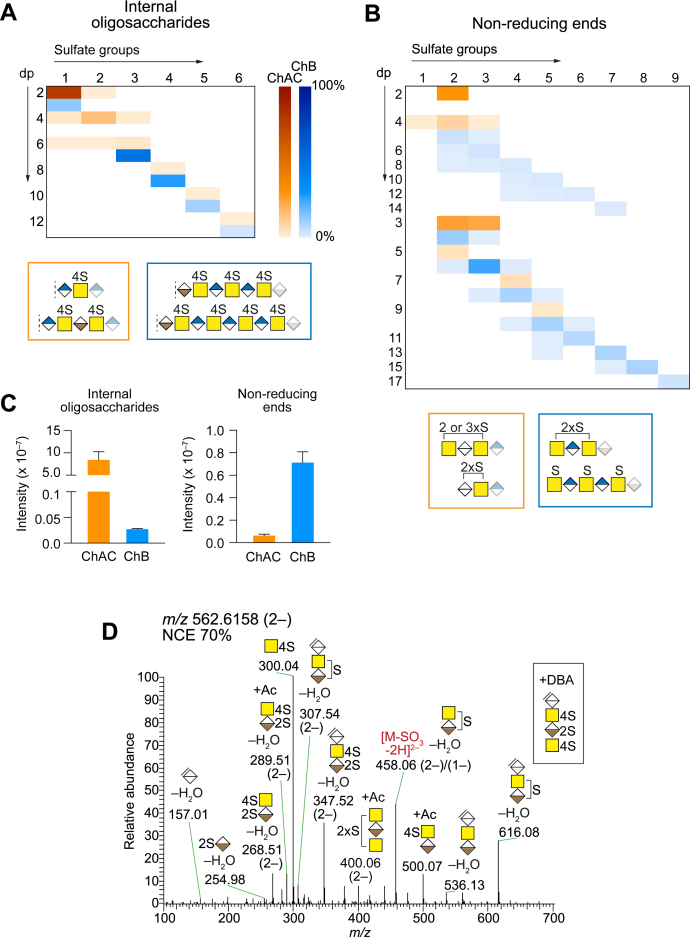
**Internal oligosaccharide and nonreducing end CS/DS from INS-1832/13 cells.***A* and *B*, heatmap summary of internal oligosaccharides (*A*) and nonreducing ends (*B*) after chondroitinase AC (ChAC, *orange*) and chondroitinase B (ChB, *blue*) depolymerizations. The intensities of the different structures were determined using an automated search routine, and the two structures with the highest intensities after each depolymerization are displayed in the boxes underneath each heatmap (ChAC, *orange*; ChB, *blue*). *C*, total intensities of internal oligosaccharides (*left*) and NREs (*right*) after ChAC (*orange*) and ChB (*blue*) depolymerizations (mean ± SD). *D*, HCD-MS^2^ spectrum of the [M+DBA-3H]^2–^ precursor ion at *m/z* 562.6158 at NCE 70% corresponding to Δdp4S3, assigning the additional sulfate group to the internal IdoA residue. Mass accuracies of precursor and fragment ions are found in supplemental Tables S1 and S2. dp, degree of polymerization.

To further extend the structural analysis of the GAGs, the internal oligosaccharides and NREs generated after chondroitinase AC and B depolymerizations were identified and relatively quantified. The internal oligosaccharides ranged from Δdp2S1 to Δdp12S6 and carried, on average, one sulfate group per disaccharide (Fig. 3*A*, and supplemental Fig. S10, *A* and *B*). The NREs ranged from dp2S2 to dp17S9 and were identified with both terminal HexA residues (even-numbered dp) and GalNAc residues (uneven-numbered dp). Similarly to the internal oligosaccharides, the NREs carried one sulfate group per GalNAc residue (Fig. 3*B*). As expected, the MS^2^ spectra of internal oligosaccharides and NREs did not differ between those generated from PG-derived GAGs (Fig. 3*D* and supplemental Fig. S10) and those generated from xyloside-primed GAGs (14).

PGs are typically divided into CSPGs and HSPGs (PGs carrying heparan sulfate (HS), another class of GAGs), but whether the CSPGs may contain IdoA residues and instead be termed CS/DSPGs is generally not considered. The chondroitinase B depolymerization of the INS-1832/13 sample resulted in the generation of internal oligosaccharides and NREs, indicating that there are indeed IdoA residues present in the GAGs (Fig. 3, *A* and B), and thereby the PGs may be considered as CS/DSPGs. However, since the majority of the CS/DS was depolymerized to disaccharides upon chondroitinase AC depolymerization (∼78%) (Fig. 3*A*), and the total intensity of the internal oligosaccharides after chondroitinase AC depolymerization was considerably higher than after chondroitinase B depolymerization (Fig. 3*C*), it was concluded that the CS/DS comprised primarily GlcA-containing disaccharides, or CS motifs. In addition to the disaccharides generated after chondroitinase AC depolymerization, Δdp4S2 was observed (∼16%), and only small amounts of larger structures (in total <4%), suggesting that the IdoA is primarily found as single IdoA-containing disaccharide units within the CS/DS and to a much lesser extent as consecutive IdoA-containing disaccharide units, or DS motifs. The MS^2^ data showed that the Δdp2S1 disaccharide obtained after chondroitinase AC depolymerization corresponded to ΔHexAGalNAc4S (supplemental Fig. S10*C*), which is in agreement with the chondroitinase ABC-generated data. Additional sulfate in Δdp4S3 was present as 2-*O*-sulfation of IdoA after chondroitinase AC depolymerization (Fig. 3*D*), which is also in agreement with the chondroitinase ABC-generated Δdp2S2 disaccharide structure (Fig. 2*C*).

The general sulfation pattern of the NREs did not deviate from that of the internal oligosaccharides, since also the NREs appeared to carry primarily one sulfate group per GalNAc residue (Fig. 3*B* and supplemental Fig. S10*E*). Chondroitinase AC depolymerization resulted mainly in di- and trisaccharide NREs (Fig. 3*B*), whereas chondroitinase B depolymerization resulted in NREs of a wider range of lengths; mainly dp3S2 (∼11%), dp5S3 (25%), dp7S4, dp9S5, dp13S7, and dp15S8 (all >8% each). The higher total intensity of NRE structures generated from chondroitinase B depolymerization than from chondroitinase AC depolymerization suggests that single IdoA-containing disaccharide units are preferentially distributed subterminally of these CS/DS chains with a varying distance of dp3–dp17 from the GAG terminals (Fig. 3*C*). However, the level of ionization of the different products may also contribute to the apparent difference in intensities, since the products after chondroitinase B depolymerization were in general considerably longer and more sulfated and thereby more likely to ionize better than those generated after chondroitinase AC depolymerization.

### Proteoglycan-Derived Linkage Regions

CgA from INS-1832/13 cells is GAGylated at Ser-433 in the peptide sequence KEEEGSANR (M.N., unpublished results). Pronase digestion specifically aims to leave only the Ser residue or just a few more amino acids linked to the GAG linkage region. In agreement with this, our data showed that Ser, Gly-Ser, Ser-Ala, and Ser-Ala-Asn were the most common amino acid compositions of the linkage region glycopeptides and that these variants eluted approximately at the same time (supplemental Fig. S11*A*). In addition, Glu-Gly-Ser and Glu-Glu-Gly-Ser glycopeptides were observed, although these eluted somewhat later (supplemental Table S3). The observed glycopeptide variants were all in accordance with the expected ones for the CgA sequence, and they were all included for each potential linkage region glycan product in the automated search. IAPP, the other and less abundant secreted CSPG identified from INS-1832/13 cells, was reported to be GAGylated at Ser-28 (M.N., unpublished results). Due to the virtual absence of precursor and fragment ion masses diagnostic for IAPP-related glycopeptide sequences, such as Gly-Val-Ser and Ser-Gly-Thr, our current data further support the notion that CgA is the main CS(/DS)PG produced by INS-1832/13 cells.

**Fig. 4:**
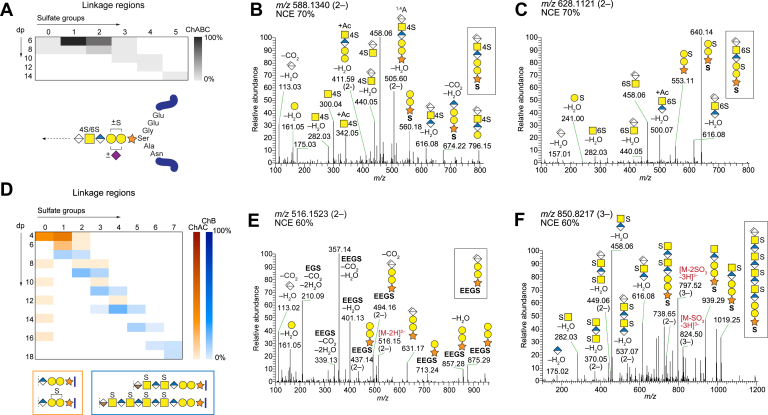
**CS/DS linkage regions from INS-1832/13 cells**. *A*, heatmap summary of the linkage region variants generated after chondroitinase ABC (ChABC; *gray*) depolymerization (*top*) and an illustration displaying observed modifications and peptide variants (*bottom*). *B* and *C*, HCD-MS^2^ spectra of linkage region hexasaccharides at *m/z* 588.1340 (2–) corresponding to ΔL6S1Ser (S) (*B*), and at *m/z* 628.1121 (2–) corresponding to ΔL6S2S (*C*), both at NCE 70%. *D*, heatmap summary of the linkage region variants generated after chondroitinase AC (ChAC; *orange*) and chondroitinase B (ChB; *blue*) depolymerizations. The two most common structures after each depolymerization are displayed in the boxes (ChAC, *orange*; ChB, *blue*) underneath the heatmap. *E*, HCD-MS^2^ spectrum at *m/z* 516.1523 (2–) at NCE 60% corresponding to the main linkage region variant generated after chondroitinase AC depolymerization, ΔL4S0Ser-Gly-Glu-Glu (**EEGS**). *F*, HCD-MS^2^ spectrum at *m/z* 850.8217 (3–) at NCE 60% corresponding to the main linkage region variant generated after chondroitinase B depolymerization, ΔL12S4S. Mass accuracies of precursor and fragment ions are found in supplemental Tables S1 and S2.

As expected from the specificity of chondroitinase ABC, hexasaccharide linkage regions (ΔL6) were the predominant linkage region variants observed after depolymerization (∼90%); however, some ΔL8–ΔL14 linkage region variants were also detected. The ΔL6 linkage regions were observed as nonsulfated, mono- and disulfated and sialylated variants (Fig. 4, *A*–*C* and supplemental Figs. S11).

Since the linkage regions constitute the essential structural difference between PG-derived GAGs and the previously described xyloside-primed GAGs, the linkage region fragmentation characteristics were studied in further detail. Fragment ions unique for the PG-derived linkage regions were observed, such as *m/z* 553.11 corresponding to Gal-Gal-Xyl(+S), where the H_2_O was retained on the glycan fragment ion rather than on the amino acid or peptide part (Fig. 4, supplemental Figs. S11 and S12). Unique cross-ring fragment ions were also observed, including *m/z* 505.60 (2–) corresponding to ΔHexA-GalNAcS-GlcA-Gal-Gal(+C_3_H_4_O_2_; 72.02 u) (Fig. 4*B*), plausibly arising from ^*1,4*^*A*-ion cross-ring fragmentation of the Xyl residue. In addition, the fragment ion at *m/z* 310.05 (2–) corresponding to ΔHexA-Gal-Gal(+S+Ac; Ac = 42.01 u) indicates ^*2,4*^*A*-ion cross-ring fragmentation of the Xyl residue (supplemental Fig. S12, *G* and *H*), in contrast to the previously described ^*0,2*^*X*-ions (14). The ^*0,2*^*X*-ion fragment ions, which also result in +Ac, arise from cross-ring fragmentation of the ΔHexA residue. For the linkage region glycopeptides constituting di- and tripeptides, the fragment ions included those corresponding to the amino acids as single units rather than stepwise peptide fragmentation that would enable peptide sequencing. However, for linkage regions with peptides larger than three amino acids, the MS^2^ spectra contained single peptide backbone fragment ions, which enabled *de novo* peptide sequencing (Fig. 4*E* and supplemental Fig. S12). Distinction between 4-*O*- and 6-*O*-sulfation of the GalNAc residues of the linkage region hexasaccharide was performed based on the relative distribution of the fragment ions at *m/z* 282.03 and *m/z* 300.04, as previously described (Figs. 4 and supplemental Fig. S11) (14, 39).

The orthogonal depolymerizations using chondroitinases AC and B resulted in linkage region variants of different lengths; after chondroitinase AC depolymerization, tetrasaccharides (ΔL4S0 and (ΔL4S1) were expected (14) and found to be the major products (∼76%) (Fig. 4, *D* and *E*), whereas after chondroitinase B depolymerization, the linkage region variants spanned more evenly over a wider size range (ΔL6S1–ΔL18S7) of which the major variant was the dodecasaccharide, ΔL12S4 (∼19%) (Fig. 4*F*). The linkage region variants appeared to carry on average one sulfate group per GalNAc residue, yet, small amounts of nonsulfated ΔL8 to ΔL16 linkage regions (in total <6%) were observed after chondroitinase AC depolymerization. Similarly to bikunin and decorin from human and bovine sources (7, 8, 39), one of the two Gal residues in the linkage regions was found carrying an *N*-acetylneuraminic acid (Neu5Ac) residue (∼4%) (supplemental Figs. S11 and S12) (M.N., unpublished results).

### Intact CS GAGs

**Fig. 5:**
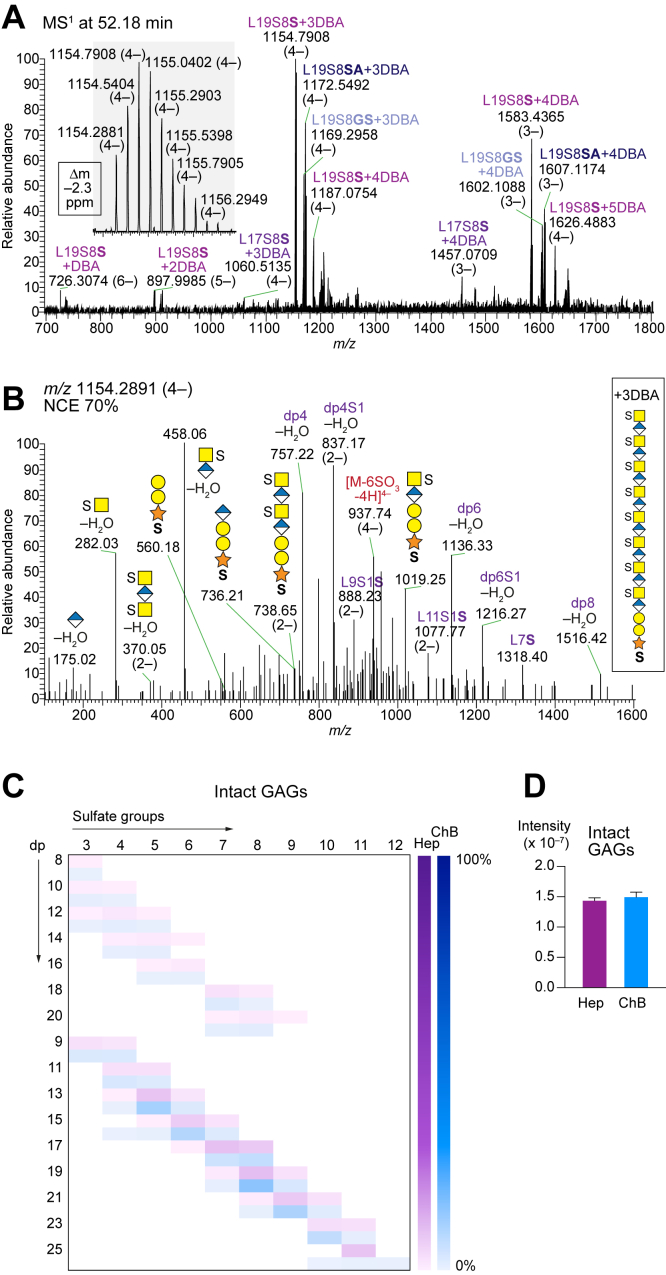
**Intact CS GAGs from INS-1832/13 cells.***A*, MS^1^ spectrum at 52.18 min displays variants of intact CS L19S8 precursor ions and charge state distribution thereof. The *inset* shows the isotopic pattern of the [M+3DBA-7H]^4–^ precursor ion at *m/z* 1154.2881 and the monoisotopic mass accuracy (Δm –2.3 ppm) from the theoretical mass. *B*, HCD-MS^2^ spectrum at *m/z* 1154.2891 (4–) where important fragment ions are annotated to confirm the assignment in (*A*). *C*, heatmap summary of intact CS GAGs after heparinase (Hep; *purple*) and chondroitinase B (ChB; *blue*) depolymerizations. *D*, total intensity comparison of intact GAGs obtained after heparinase (Hep; *purple*) and chondroitinase B (ChB; *blue*) depolymerizations (mean ± SD). Mass accuracies of fragment ions are found in supplemental Table S2. DBA, dibutylamine.

When performing either heparinase or chondroitinase B depolymerizations, several precursor ions corresponding to nondepolymerized GAG structures, or intact GAGs, of the Ser, Gly-Ser, Ser-Ala variants were identified (Fig. 5*A* and Supplemental Fig. S13). Manual interpretation of the MS^2^ spectra supported that the products were nondepolymerized structures; for example, the [M+3DBA-7H]^4–^ precursor ion at *m/z* 1154.2891 corresponding to L19S8Ser contained fragment ions at *m/z* 1019.25 and *m/z* 738.65 (2–) corresponding to GalNAc-GlcA-Gal-Gal-Xyl-*O*-Ser(+S) and GalNAc-GlcA-GalNAc-GlcA-Gal-Gal-Xyl-*O*-Ser(+2xS), respectively (Fig. 5*B*). By generating a list of monoisotopic masses of potential intact GAGs and applying our search routine, identification of a wide range of intact GAG structures was conceivable. The sizes ranged from L8S3 to L25S12 (Fig. 5*C*), and the relative abundances of the different structural variants were similar after the heparinase and chondroitinase B depolymerizations. This implies that rather short GAG chains entirely of the CS character were present in addition to the CS/DS GAG chains. Furthermore, the total intensities of the intact products remaining after the depolymerizations were similar (Fig. 5*D*), suggesting that the CS/DS GAGs were not included in the population of CS GAGs as this would have resulted in a higher intensity of the structures generated after heparinase depolymerization than after chondroitinase B depolymerization. Thus, the population of CS/DS GAGs is most likely longer than the intact CS GAGs observed here and not identified after heparinase depolymerization due to the present chain length detection limit.

### Structural Summary

**Fig. 6:**
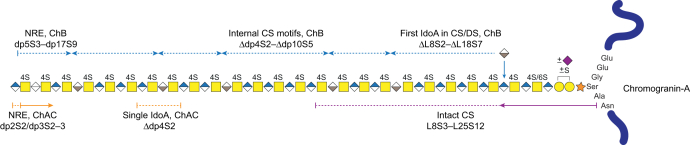
**Structural summary of the CS/DS GAG of chromogranin-A from INS-1832/13 cells.** Schematic CS/DS structure estimated based on the compositional profiling and MS^2^ data of NREs, internal oligosaccharides, linkage regions, and intact structures obtained after the different depolymerizations as illustrated by the lines and *arrows* above and below the CS/DS structure (chondroitinase AC, ChAC, *orange*; chondroitinase B, ChB, *blue*; heparinase and ChB, *purple*). The average GAG length is based on western blot data (M.N., unpublished results).

Since CgA appears as the dominating PG produced by INS-1832/13 cells (M.N., unpublished results), an overall CS/DS GAG structure of CgA from INS-1832/13 cells may be concluded based on our data (Fig. 6). Starting from the linkage region, peptide variants that covered six amino acids of CgA were identified, as well as sulfation and sialylation modifications of the linkage region tetrasaccharide. The first IdoA residue, when present, appeared a couple of disaccharides away from the linkage region tetrasaccharide, and the elongated GAG chain comprised longer CS motifs intersected by single IdoA residues, instead of proper DS motifs comprising consecutive IdoA residues. IdoA residues appeared rather toward the NRE and the reducing end, or the linkage region, than as a part of the internal oligosaccharides. The terminal monosaccharide residues of the NREs comprised both GalNAc and HexA residues. In general, the GAG chain carried one sulfate group per GalNAc residue and the sulfation occurred at position 4. The average chain length, ∼12.5 kDa, corresponding to ∼dp54 (determined using a molecular weight of 459 Da per disaccharide), was estimated using western blot data (M.N., unpublished results) and further supports our hypothesis that the CS/DS and CS GAG subpopulations differ in chain length. Altogether, the CgA CS/DS chain from INS-1832/13 cells appears to have a rather simple structure in terms of sulfation, but heterogeneous with respect to chain length, IdoA distribution, linkage region modifications, and NRE residues.

## Discussion

Development of methods in glycomics is essential to enable and refine structural analysis of the complex GAGs and GAGomes. Here, we have introduced the glycosaminoglycan domain mapping, GAGDoMa, of PG-derived GAGs. The approach enabled compositional profiling, relative quantification, and detailed MS^2^ characterization of NREs, internal oligosaccharides, and linkage regions of PG-derived CS/DS. To facilitate data interpretation and enable the use of replicates to provide a more reliable relative quantification, we introduced an automated search routine. Other software tools available for glycan characterization and quantification, such as GlycReSoft (40, 41), have been developed over the years to provide more robust and powerful analyses. However, in this study we opted for a fairly simple approach for the relative quantification of the signal intensity: the LC-MS peaks were detected and quantified by the fast Minora Feature Detector node in Proteome Discoverer, and the resulting peaks were explicitly matched to the expected glycan masses. Taken together, our approach is efficient, and the instrument setup and data interpretation are easily implemented into most proteomics laboratories.

During the protocol development for isolation and purification of PG-derived GAGs, we discovered that the presence of oligonucleotides and HA in the sample preparations obstructed not only the data interpretation, but also the activity of chondroitinases ABC and AC. This reduction in enzyme activity is essential to keep in mind when preparing GAG samples for structural analysis, since most of the current approaches are highly dependent on the efficiencies of the GAG depolymerizing enzymes (3, 4). In addition to external factors that may influence the activities of these enzymes, the enzymes themselves appear to have different activities depending on the manufacturer (42). Thus, it is important to use an analytical method, such as the one presented here (Fig. 2*A*), where the completion of the depolymerization processes can be followed.

Future refinement of the GAGDoMa method includes exploring the size limitation of the GAGs using GAG standards of well-defined lengths and sulfation levels. In addition, GAG standards would aid in resolving longer and more complex structures and improve the relative quantification by, for example, allowing to take the level of ionization of different products into consideration. Future studies also aim to include structural characterization of HS GAGs. HS is another interesting class of GAGs not least from a structural aspect due to the possibility of *N*-sulfation of the GlcNAc residues. Furthermore, the specificities of the existing HS depolymerizing enzymes may allow for domain mapping of HSPGs using the GAGDoMa approach.

The CS/DS GAG structure of CgA from INS-1832/13 cells appeared relatively simple since it primarily comprised GlcAGalNAc4S disaccharide units. However, the structure was not strictly uniform as it contained single IdoA residues throughout the chain and additional 2-*O*-sulfations of IdoA, or GlcA, residues. The first IdoA residue detected in the CS/DS chain occurred 1–7 disaccharide units away from the linkage region tetrasaccharide, instead of only directly after the first GalNAc residue. Whether this impacts the level and distribution of IdoA residues in the downstream GAG chain remains to be elucidated. The Neu5Ac-containing linkage region variants were detected only after chondroitinase ABC and AC depolymerizations, suggesting that the Neu5Ac modification may be involved in directing the biosynthesis toward CS or CS/DS structures. Only the secreted CgA was studied here, arguing that the described structures are not products of ongoing GAG biosynthesis, which may mislead the structural conclusions as to the level of heterogeneity. Nevertheless, it has to be taken into account that GAGs from other core proteins than CgA may be present in small amounts. The identification of both intact CS GAGs and CS/DS copolymeric GAGs implies that there are different subpopulations present, a matter touched upon previously in relation to the xyloside-primed GAGs (14). Since we speculate that the secreted GAGs result from biosynthetic completion, the different subpopulations may contribute to different structure-related functions. However, further studies are required to draw conclusions regarding this complex matter.

CgA is a recently established part-time hybrid CS- and HSPG (M.N., unpublished results) that may serve as a key player in secretory granule formation during, for example, insulin secretion (15, 43). For the first time, we can now conclude that the CS of CgA produced by INS-1832/13 cells does contain IdoA to some extent, and CgA may therefore be considered as a hybrid CS-, CS/DS-, and HSPG. Whether the structure may be influenced by different culture conditions remains to be elucidated; nevertheless, the GAGDoMa approach will allow for such studies.

One may only speculate on the biological impact of the current findings, especially since there is yet no translation from specific GAG structures of CgA to functions. A general collection of structural data will create a foundation on which the relevance of certain or overall structural features may eventually be concluded. The presented data are expected to be a snapshot of the INS-1832/13 cell GAGome or the repertoire of GAG structures for CgA. However, the structures may be influenced by different stimuli (44) and, for example, age, or passages for cell cultures, *etc*. Furthermore, it is unlikely to acquire one definite or absolute structure from all the GAGs from a biological sample of interest, since the samples *per se* contribute to the structural data, and to address a certain biological question, ideally well-defined conditions should be compared. To aim for one absolute structural variant of the apparent GAGs may inadvertently hamper the possibilities of discovering new structural features; thus, -omics-based strategies as exemplified in this work may become of greater relevance for understanding complex biological processes.

The GAGDoMa approach presented here represents an important methodological step in structural glycobiology since it enables studies of GAGomes and specific PGs at a high level of details. Furthermore, it may be used for diagnostic purposes, aid in understanding of pathological mechanisms, and eventually decode novel structure–function relationships of GAGs and PGs.

## Data Availability

MS data have been deposited to the ProteomeXchange consortium *via* the PRIDE partner repository (45) with the data set identifier PXD023018.

## Conflict of interest

The authors declare no competing interests.
