# Supplementary material for: Domain Mapping of Chondroitin/Dermatan Sulfate Glycosaminoglycans Enables Structural Characterization of Proteoglycans
Source: Mol Cell Proteomics. 2021 Mar 20;20:100074. doi: 10.1016/j.mcpro.2021.100074 (PMC8724862; doi:10.1016/j.mcpro.2021.100074)
Supplement: Supplemental Figures [file mmc1.pdf]

Supplemental data

Domain mapping of chondroitin/dermatan sulfate glycosaminoglycans enables structural characterization of proteoglycans

Andrea Persson, Mahnaz Nikpour, Egor Vorontsov, Jonas Nilsson, and Göran Larson

Figures S1–S13

A

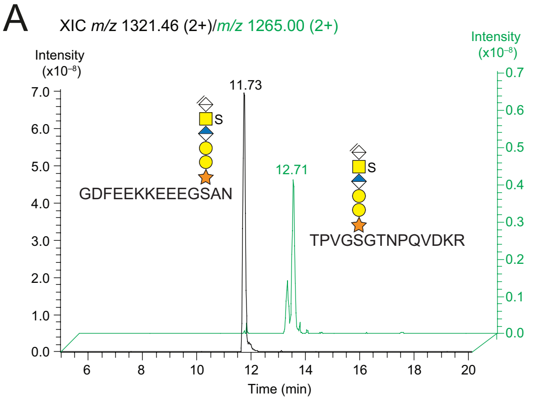

B

File number	PG	Sulfate groups	m/z (3+)	AUC	m/z (2+)	AUC	Total	% of total
180521_30	CgA	0	854.65	132901879	1281.48	28803193		
		1	881.32	2780348181	1321.46	4820580746		
		2	907.96	143919642	1361.44	413689240	8320242881	93
	IAPP	0	817.32	100196138	1225.03	4196397		
		1	843.67	115634680	1265.00	243723389		
		2	870.32	38751654	1304.98	76504627	579006885	7
						**Sum**	8899249766	
180521_36	CgA	0	854.65	96996369	1281.48	16632794		
		1	881.32	1505487490	1321.46	2310381563		
		2	907.96	69937694	1361.44	353872734	4353308644	94
	IAPP	0	817.32	48824401	1225.03	–		
		1	843.67	56084205	1265.00	122382599		
		2	870.32	21162843	1304.98	19586571	268040619	6
						**Sum**	4621349263	

Figure S1. Relative abundance of the secreted PGs from INS-1 832/13 cells. *A*, Extracted ion chromatograms (XICs) at *m/z* 1321.46 (2+) corresponding to the GDFEEKKEEEGSAN glycopeptide from chromogranin-A (CgA) modified with a monosulfated hexasaccharide, and at *m/z* 1265.00 (2+) corresponding to the TPVGSGTNPQVDKR glycopeptide from islet amyloid polypeptide (IAPP) modified with a monosulfated hexasaccharide. *B*, Summarizing table of the relative abundances of the glycopeptides in (*A*) non-modified or modified with one or two sulfate groups based on the area under the curves (AUCs). The data display that CgA is the dominating secreted PG from INS-1 832/13 cells. The two files are samples replicates. MS data have been deposited to the ProteomeXchange consortium via the PRIDE partner repository[1] with the dataset identifier PXD024230.

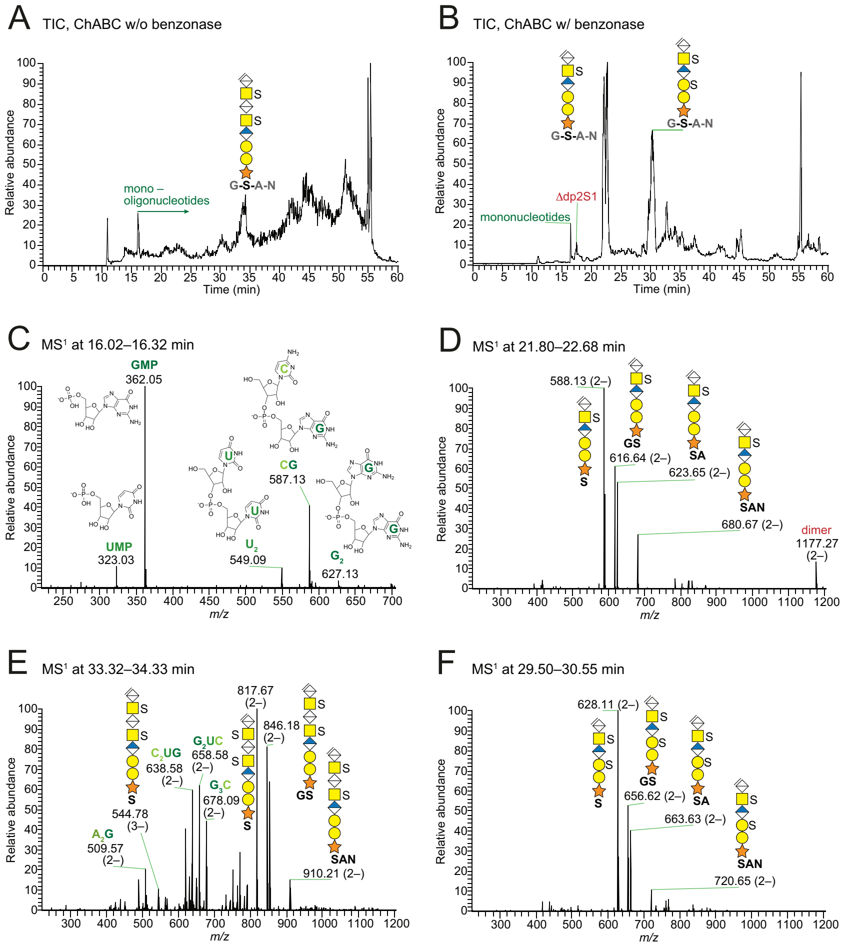

Figure S2. Mono- and oligonucleotides in GAG sample preparation from INS-1 832/13 cells and impact of benzonase digestion on chondroitinase ABC depolymerization. *A*,*B*, Total ion chromatograms (TICs) of GAGs from INS-1 832/13 cells without (w/o; *A*) and with (w/; *B*) benzonase digestion prior chondroitinase ABC depolymerization. *C*,*E*, MS^1 spectra of the main peaks in (*A*) at 16.02–16.32 min (*C*) and at 33.32–34.33 min (*E*), corresponding to RNA-derived mono- and oligonucleotides and linkage region octasaccharides, respectively. *D*,*F*, MS^1 spectra of two of the main peaks in (*B*) at 21.80–22.68 min (*D*) and at 29.50–30.55 min (*F*), corresponding to mono- and disulfated linkage region hexasaccharides, respectively. MS^2 spectra of selected nucleotide-derived precursor ions are shown in Fig. S3. The presence of oligonucleotides did not only obstruct the data interpretation, but also the GAG depolymerization using chondroitinase ABC. The latter was concluded by the difference in the abundance of the expected hexasaccharide linkage region variants and the incompletely depolymerized octasaccharide linkage region variants, which without benzonase digestion were 28% and 72%, respectively, and with benzonase digestion were 96% and 4%, respectively. Raw data are found in Supplementary Table S3.

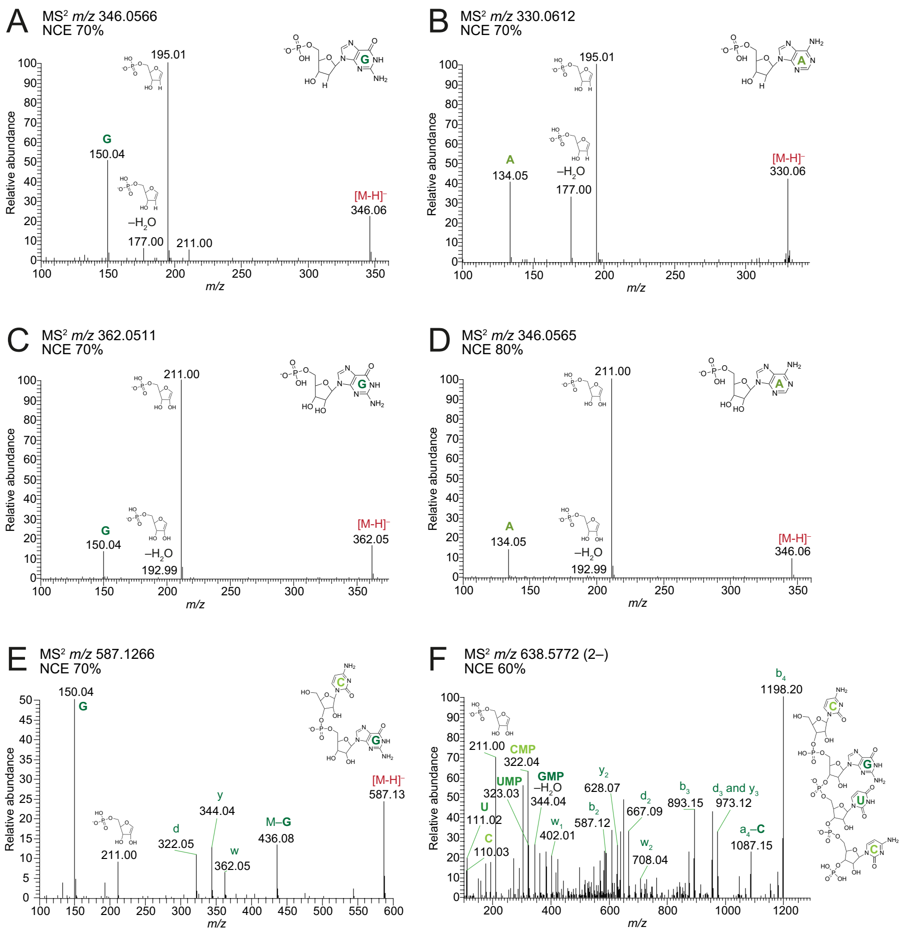

Figure S3. Characterization of mono- and oligonucleotides in GAG sample preparation from INS-1 832/13 cells. *A*–*F*, MS^2 spectra of the precursor ions at *m/z* 346.0566 corresponding to dGMP (*A*), at *m/z* 330.0612 corresponding to dAMP (*B*), at *m/z* 362.0511 corresponding to GMP (*C*), at *m/z* 346.0565 corresponding to AMP (*D*), at *m/z* 587.1266 corresponding to the CG dinucleotide (*E*), and at *m/z* 638.5772 (2–) corresponding to the CGUC tetranucleotide (*F*). Proposed annotations of fragment ions are based on the nomenclature by Closkey *et al*[2].

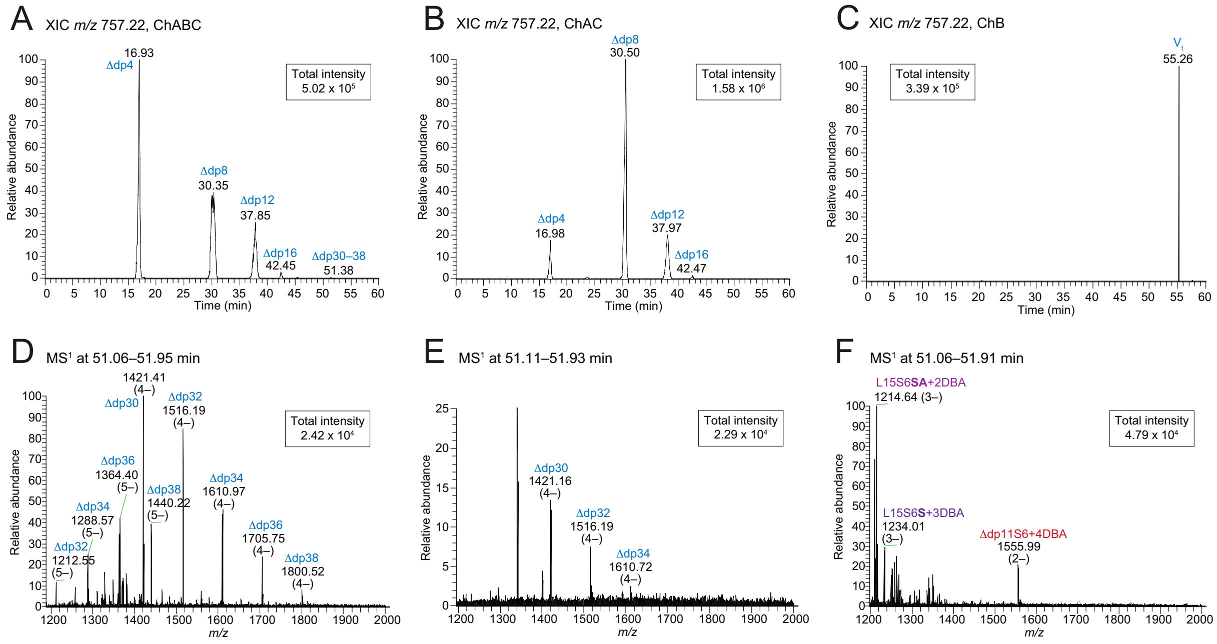

Figure S4. Non-sulfated oligosaccharides generated after chondroitinase ABC and AC depolymerizations of GAGs from INS-1 832/13 cells, but not after chondroitinase B depolymerization. *A–C*, XICs at *m/z* 757.22 (n–) corresponding to unsaturated non-sulfated oligosaccharides, Δdp4n (n=1, 2, 3…), after chondroitinase ABC (*A*), chondroitinase AC (*B*) and chondroitinase B (*C*) depolymerizations. *D–F*, MS^1 spectra at 51–52 min after chondroitinase ABC (*D*), chondroitinase AC (*E*) and chondroitinase B (*F*) depolymerizations. LC-MS was performed using a C18 column. The data display the presence of unsaturated non-sulfated oligosaccharides generated after chondroitinase ABC and AC depolymerizations, but not after chondroitinase B depolymerization. Since chondroitinases ABC and AC[3,4], but not chondroitinase B, are known to depolymerize both non-sulfated CS, chondroitin, and hyaluronic acid (HA), additional experiments were performed to determine the origin of the non-sulfated oligosaccharides (Fig. S5 and S6).

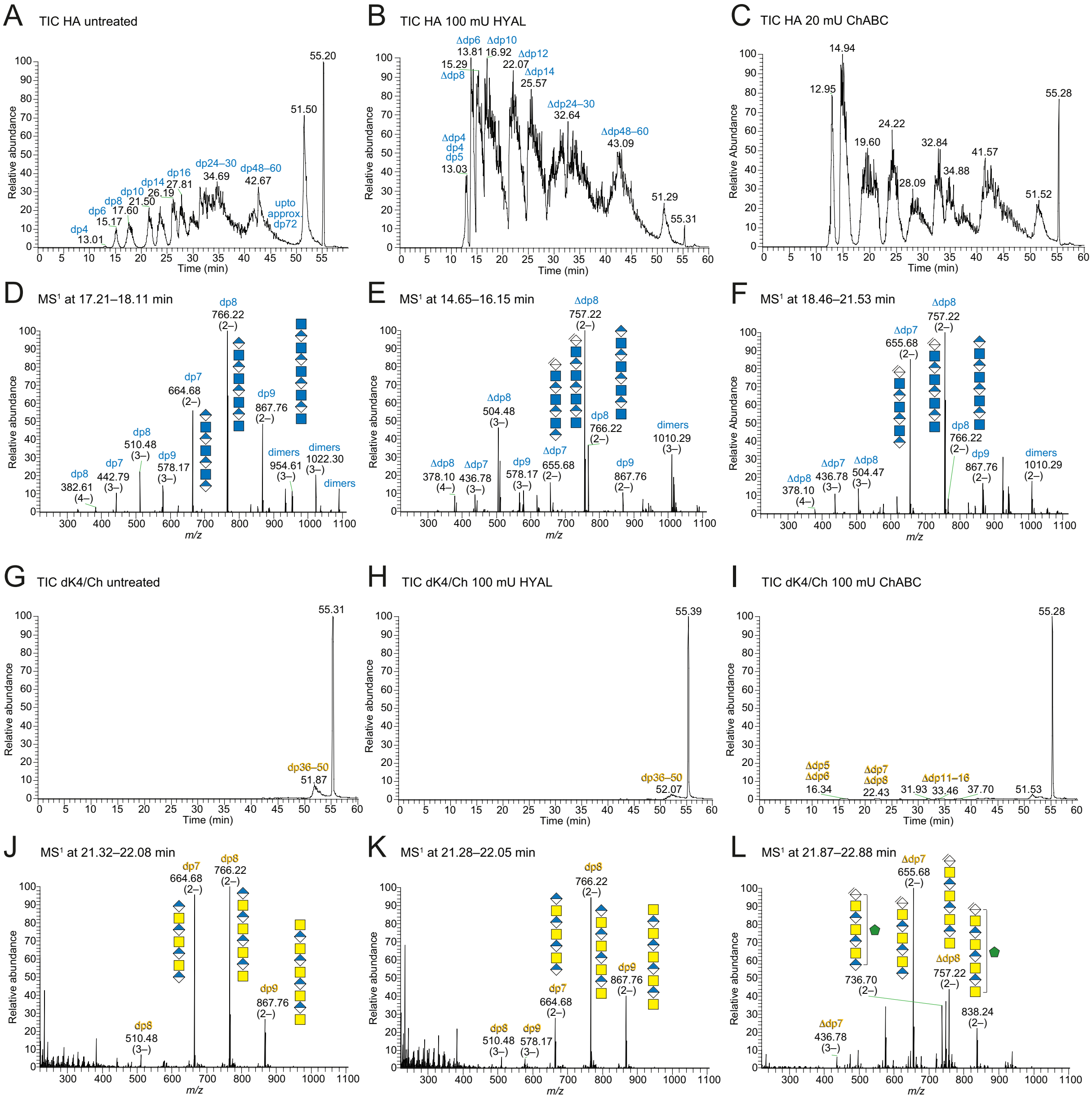

Figure S5. Susceptibility of hyaluronic acid and chondroitin to hyaluronidase and chondroitinase ABC depolymerizations. *A–C*, TICs of untreated hyaluronic acid (HA; *A*), and HA depolymerized with hyaluronidase (HYAL; *B*) or chondroitinase ABC (ChABC; *C*) display depolymerization of HA with either hyaluronidase or chondroitinase ABC. *D–F*, MS^1 spectra at 17.21–18.11 min (*D*) displaying saturated HA oligosaccharides in (*A*), at 14.65–16.15 min (*E*) displaying unsaturated and saturated HA oligosaccharides (1:0.4) in (*B*), and at 18.46–21.53 min (*F*) displaying unsaturated and saturated HA oligosaccharides (1:0.1) in (*C*). *G–I*, TICs of untreated defructosylated K4 polysaccharide/chondroitin (dK4/Ch; *G*), and dK4/Ch depolymerized with hyaluronidase (HYAL; *H*) or chondroitinase ABC (ChABC; *I*) display limited, yet some, depolymerization of dK4/Ch using chondroitinase ABC. *J–L*, MS^1 spectra at 21.32–22.18 min (*J*) displaying saturated dk4/Ch oligosaccharides in (*G*), at 21.28–22.05 min (*K*) displaying saturated HA oligosaccharides in (*H*), and at 21.87–22.88 min (*L*) displaying unsaturated dK4/Ch oligosaccharides in (*I*). LC-MS was performed

using a C4 column. The large peak appearing at ~55 min in all TICs corresponds to HA or dK4/Ch larger than those possible to resolve using the current LC system.

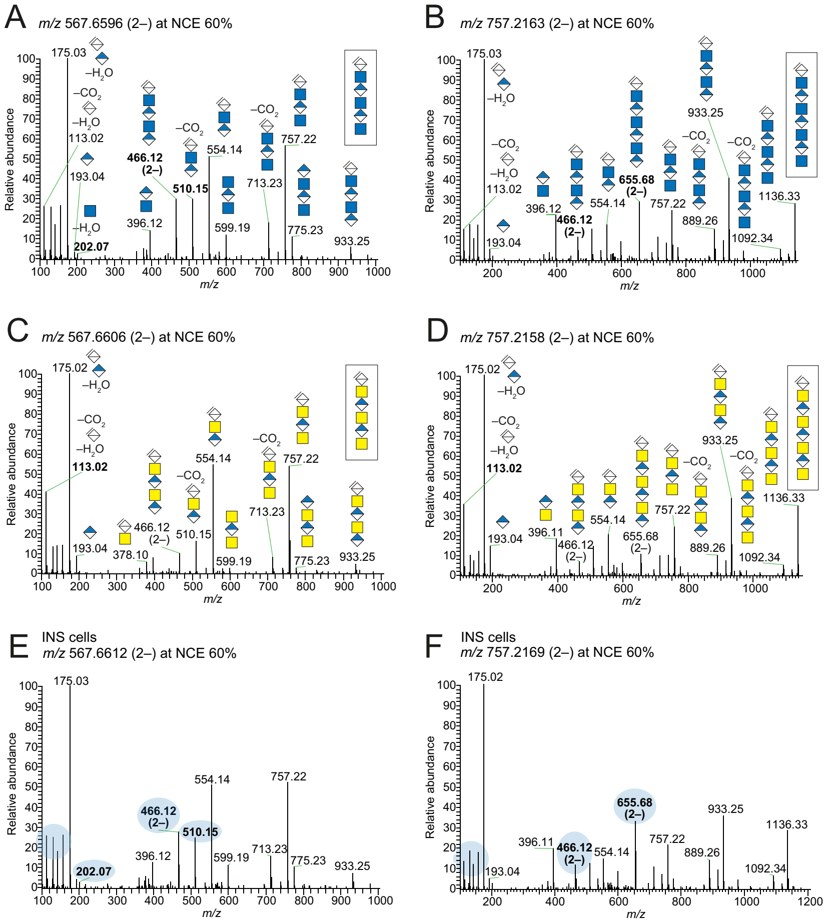

Figure S6. Distinction between hyaluronic acid and chondroitin at MS^2 level. *A–D*, MS^2 spectra of the $[M-2H]^{2-}$ precursor ions at *m/z* 567.66 (*A*,*C*) corresponding to unsaturated hexasaccharides, Δdp6, and at *m/z* 757.22 (*B*,*D*) corresponding to unsaturated octasaccharides, Δdp8 (*B*,*D*), derived from HA (*A*,*B*) and dK4/Ch (*C*,*D*). The spectra display that it is possible to distinguish between HA and dK4/Ch at MS^2 level. Important fragment ions are indicated in bold. *E*,*F*, MS^2 spectra at *m/z* 567.6612 (2–) (*E*) and at *m/z* 757.2169 (2–) (*F*) display that the non-sulfated oligosaccharides from INS-1 832/13 cells after chondroitinase ABC and AC depolymerizations correspond to HA. Important fragment ions are indicated in bold and shaded in blue.

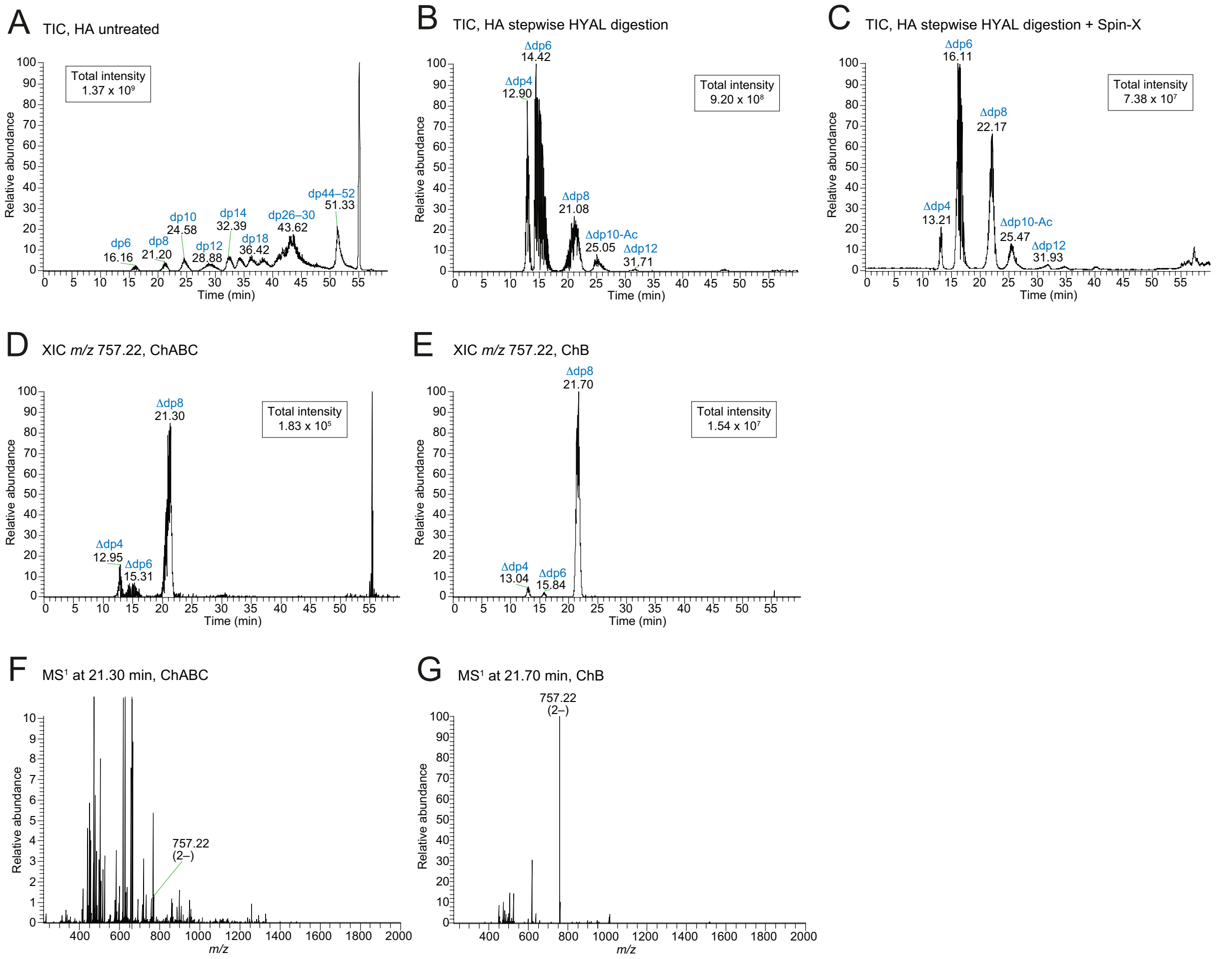

Figure S7. Hyaluronic acid removal using stepwise hyaluronidase digestion and filter columns. *A*–*C*, TICs of untreated HA (*A*), and HA subjected to stepwise hyaluronidase digestion (in total 1.2 U of hyaluronidase) without (*B*) and with (*C*) a Spin-X UF step to remove oligosaccharides. The oligosaccharide size and total intensities (boxed) of the different TICs show that the amount of HA is greatly reduced upon hyaluronidase digestion and Spin-X UF filtration. *D*,*E*, XICs at *m/z* 757.22 after chondroitinase ABC (*D*) and chondroitinase B (*E*) depolymerizations display the impact of hyaluronidase digestion. LC-MS was performed using a C4 column. Compare Fig. S7 to Fig. S4; the differences in retention times between Fig. S7 and S4 are due to the different LC columns used. *F*–*G*, MS^1 spectra display the $[M\text{-}2H]^{2-}$precursor ion at *m/z* 757.22 at 21.30 min after chondroitinase ABC depolymerization (*F*) and at 21.70 min after chondroitinase B depolymerization (*G*).

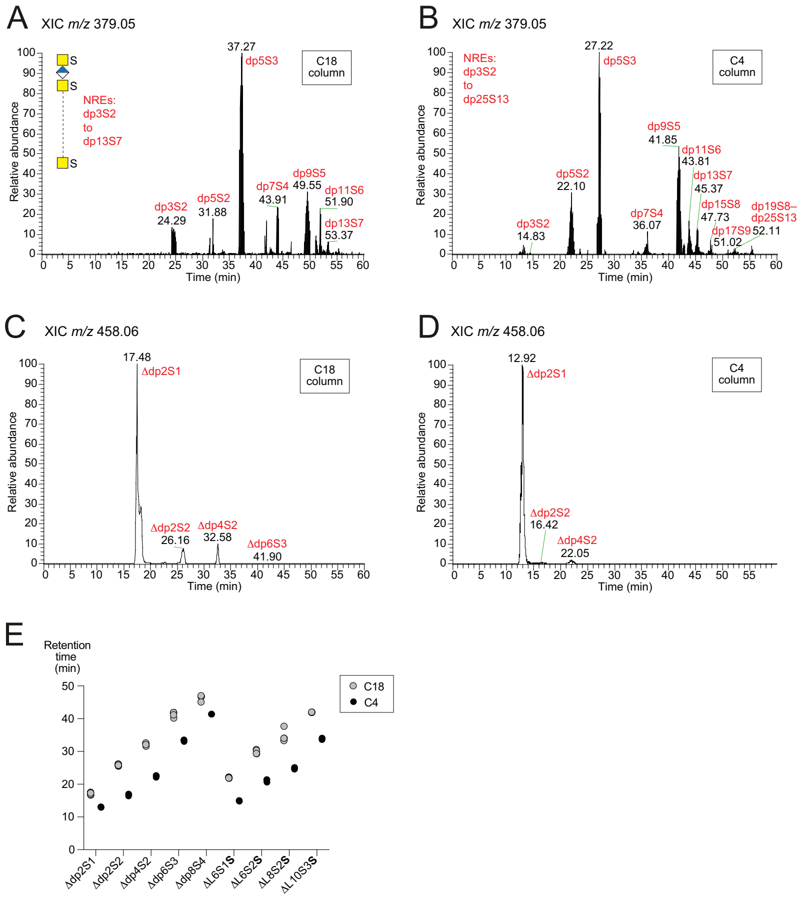

Figure S8. Comparison of oligosaccharide separation using C18 and C4 LC columns. *A*,*B*, XICs of the fragment ion at *m/z* 379.05 (2–) corresponding to dp3S2 (GalNAcHexAGalNAc+2S) display NRE oligosaccharide structures after chondroitinase B depolymerization detected using C18 (*A*) and C4 (*B*) columns. The chromatograms show that the C4 column allows detection of larger NRE structures than the C18 column. *C*,*D*, XICs at *m/z* 458.06 corresponding to oligosaccharides carrying one sulfate group per disaccharide (Δdp2nSn, n=1, 2, 3…) after chondroitinase ABC depolymerization using C18 (*C*) and C4 (*D*) columns. The higher intensities of the larger structures appearing in (*C*) as compared to (*D*) result from hyaluronidase not being included in the sample preparation. *E*, Scatter plot showing the retention times of commonly occurring oligosaccharide structures using C18 (grey) and C4 (black) columns (n=4 or 5). The graph shows highly reproducible results both for the C18 and C4 columns, and the shorter retention times obtained using the C4 column. Raw data are found in Supplementary Table S3.

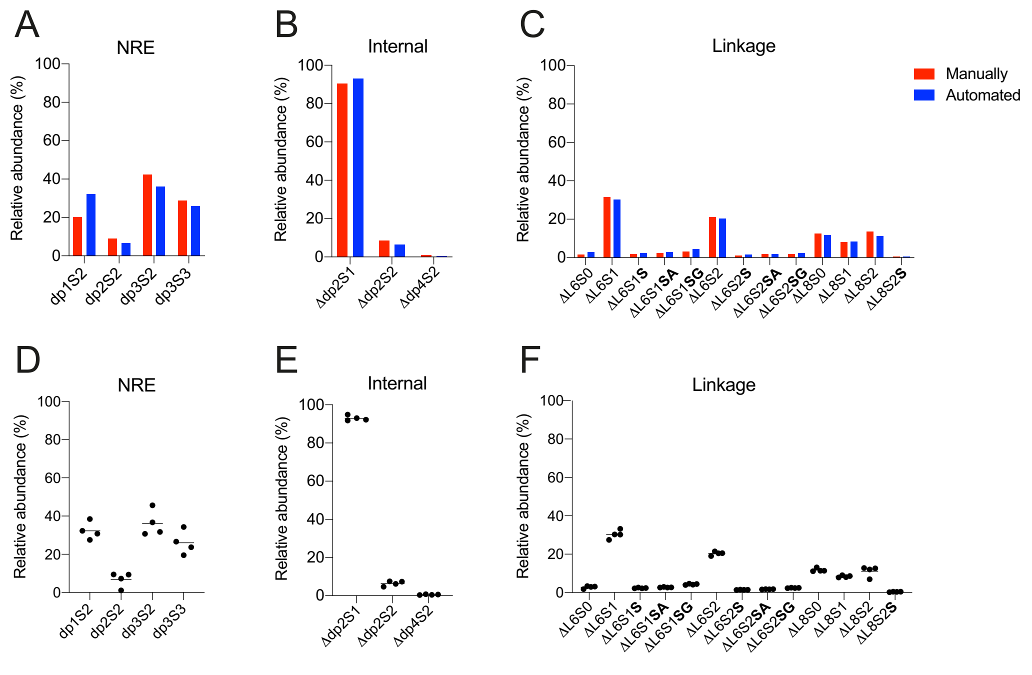

Figure S9. Verification of automated data interpretation. *A–C*, Comparison of the relative abundances of NREs (*A*), internal oligosaccharides (*B*), and linkage regions (*C*) of 4-*O*-sulfated CS after chondroitinase ABC depolymerization between data generated manually (red) and using our automated search routine (blue). *D–F*, Scatter plots displaying the reproducibility between runs of the relative abundances of NREs (*D*), internal oligosaccharides (*E*), and linkage regions (*F*) from 4-*O*-sulfated CS after chondroitinase ABC depolymerization (n=4).

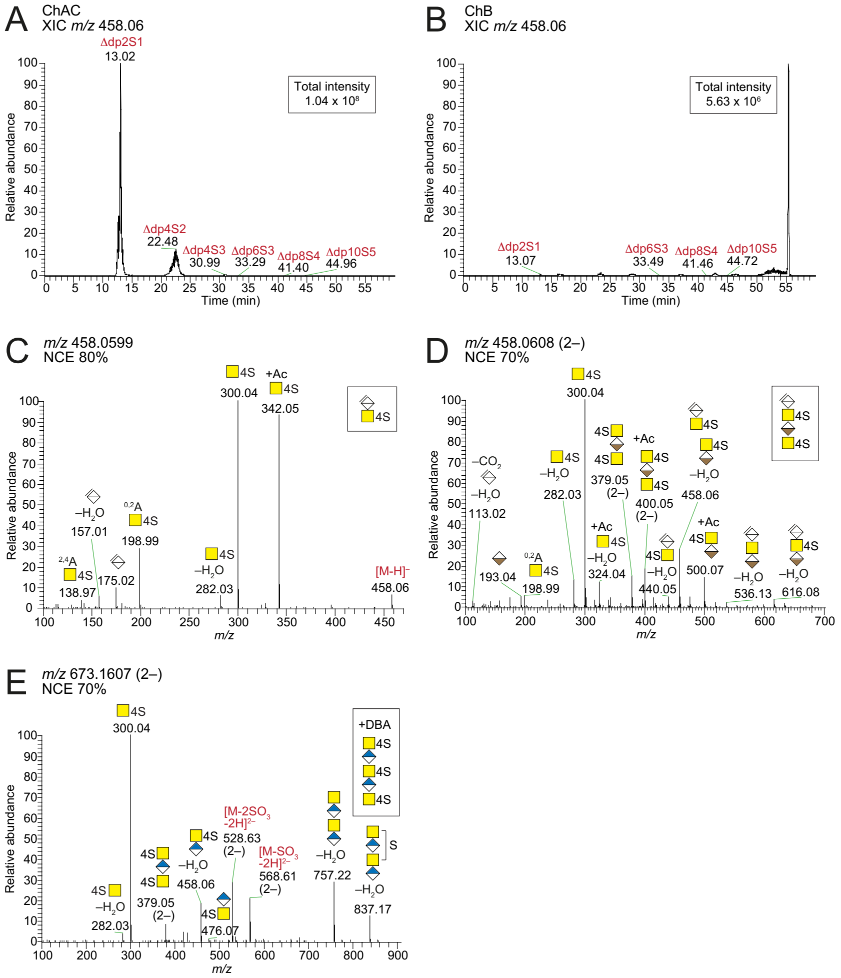

Figure S10. CS/DS oligosaccharides and NREs from INS-1 832/13 cells. *A*,*B*, XICs at m/z 458.06 corresponding to disaccharides carrying one sulfate group per disaccharide after chondroitinase AC (*A*) and chondroitinase (*B*) depolymerizations. *C*, MS^2 spectrum at *m/z* 458.0599 at NCE 80% corresponding to the ΔHexAGalNAc4S disaccharide after chondroitinase AC depolymerization. *D*, MS^2 spectrum at *m/z* 458.0608 (2–) at NCE 70% corresponding to the IdoA isomer of Δdp4S2[5] after chondroitinase AC depolymerization. *E*, MS^2 spectrum at *m/z* 673.1607 (2–) at NCE 70% corresponding to the dp5S3 NRE after chondroitinase B depolymerization.

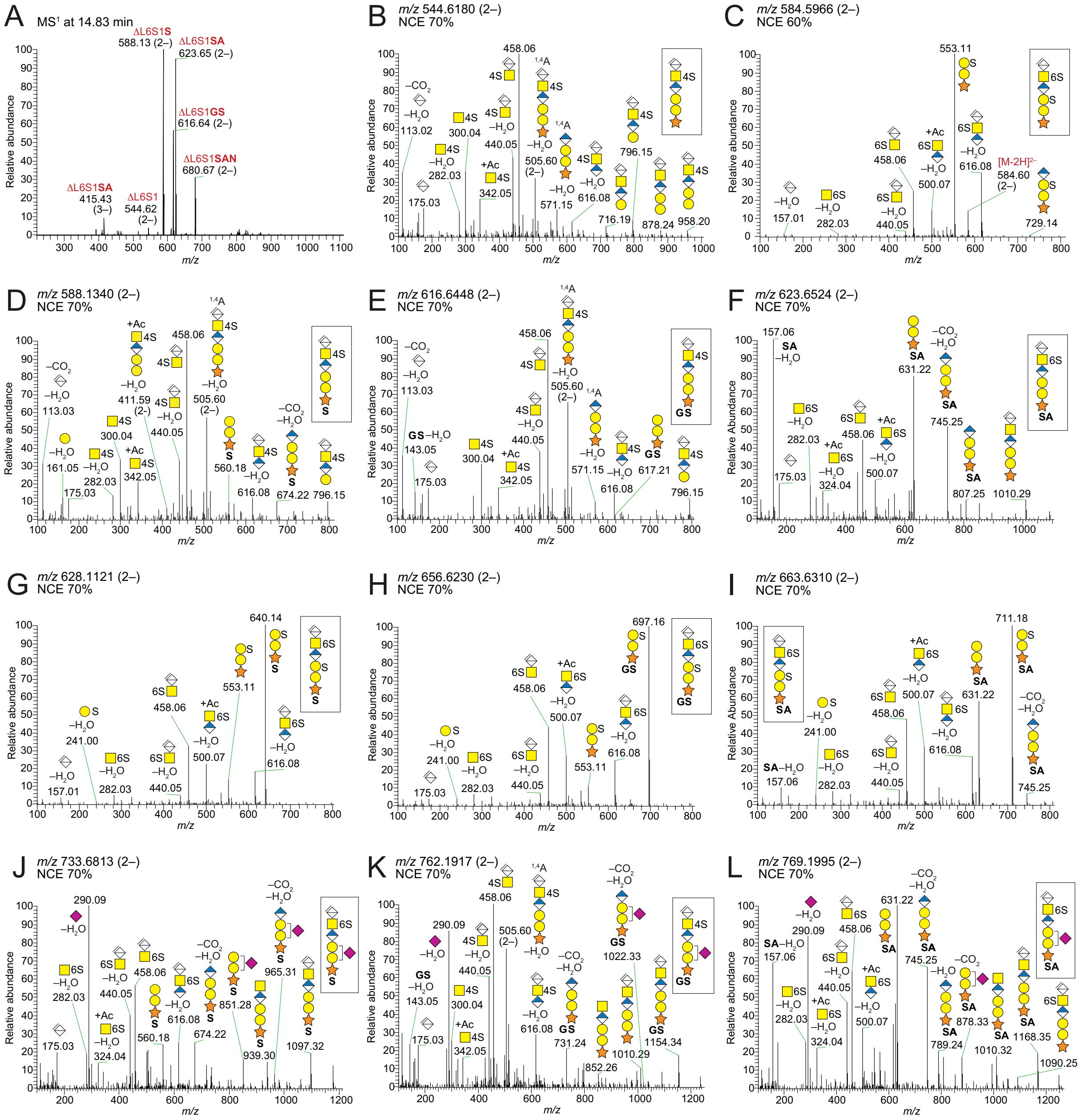

Figure S11. Linkage region hexasaccharide variants from INS-1 832/13 cells generated after chondroitinase ABC depolymerization. *A*, MS^1 spectrum at 14.83 min displaying co-eluting [M-2H]$^{2–}$ precursor ions corresponding to linkage region variants of the monosulfated hexasaccharide without or with one or two residual amino acid residues. *B*,*C*, MS^2 spectra at *m/z* 544.6180 (2–) (*B*) and at *m/z* 584.5966 (2–) (*C*) corresponding to amino acid-lacking monosulfated and disulfated linkage region hexasaccharides, respectively. *D*–*F*, MS^2 spectra at *m/z* 588.1340 (2–) (*D*), at *m/z* 616.6448 (2–) (*E*), and at *m/z* 623.6524 (2–) (*E*) corresponding to monosulfated linkage region hexasaccharides with Ser (**S**), Gly-Ser (**GS**), and Ser-Ala (**SA**) residues, respectively. *G*–*I*, MS^2 spectra at *m/z* 628.1121 (2–) (*G*), at *m/z* 656.6230 (2–) (*H*), and at *m/z* 663.6310 (2–) (*I*) corresponding to disulfated linkage region hexasaccharides with Ser, Gly-Ser, and Ser-Ala residues, respectively. *J*–*L*, MS^2 spectra at *m/z* 733.6813 (2–) (*J*), at *m/z* 762.1917 (2–) (*K*), and at *m/z* 769.1995 (2–) (*L*) corresponding to monosialylated and monosulfated linkage region hexasaccharides with Ser, Gly-Ser, and Ser-Ala

residues, respectively. NCE 70% was applied to all spectra except for the one in (*C*) where NCE 60% was applied.

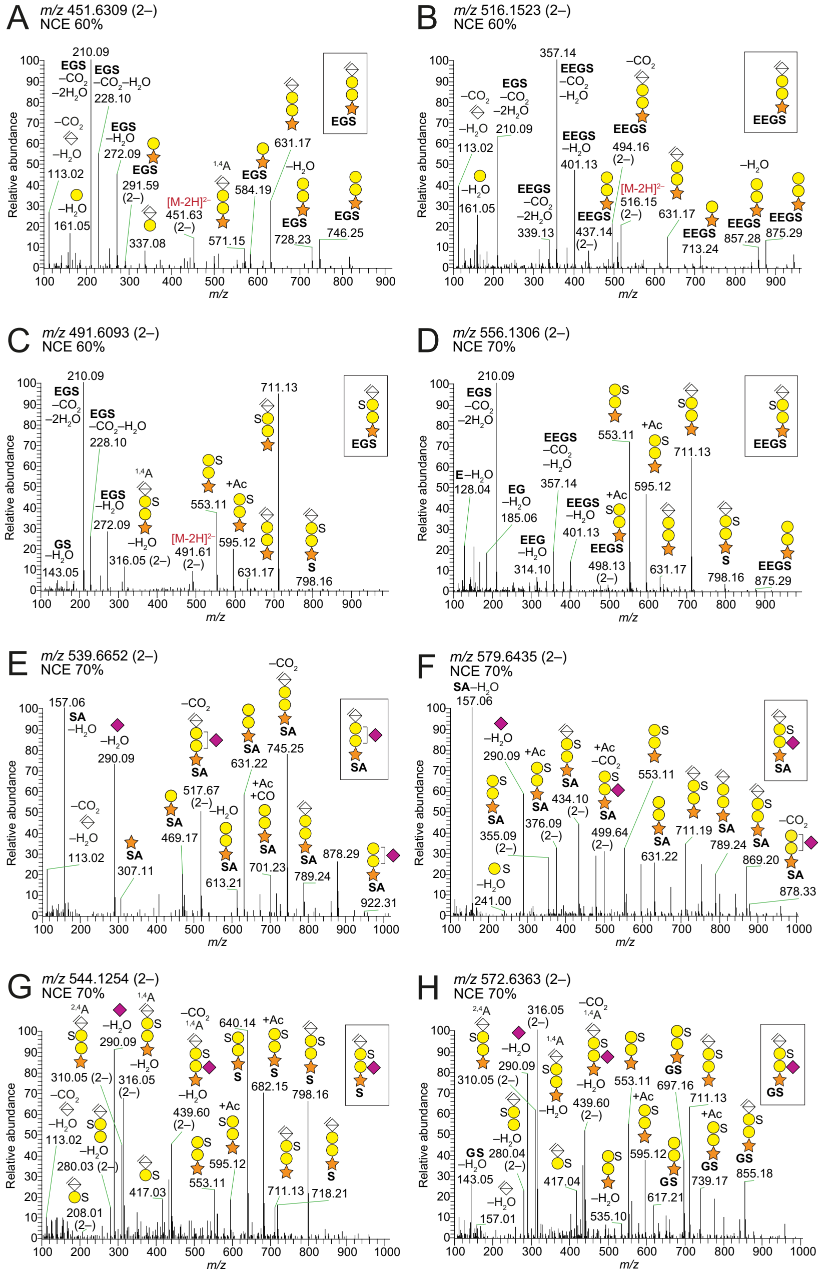

Figure S12. Linkage region tetrasaccharide variants from INS-1 832/13 cells generated after chondroitinase AC depolymerization. *A*,*B*, MS^2 spectra at *m/z* 451.6309 (2–) (*A*) and at *m/z* 516.1523 (2–) (*B*), both at NCE 60%, corresponding to the non-sulfated tetrasaccharide linkage regions with Glu-Gly-Ser (**EGS**) and Glu-Glu-Gly-Ser (**EEGS**) residues, respectively. *C*,*D*, MS^2 spectra at *m/z* 491.6093 (2–) at NCE 60% (*C*) and at *m/z* 556.1306 (2–) at NCE 70% (*D*), corresponding to monosulfated tetrasaccharide linkage regions with Glu-Gly-Ser and Glu-Glu-Gly-Ser residues, respectively. *E*, MS^2 spectrum at *m/z* 539.6652 (2–) at NCE 70%, corresponding to tetrasaccharide linkage regions carrying one *N*-acetylneuraminic acid (Neu5Ac) residue. *F–H*, MS^2 spectra at *m/z* 579.6435 (2–) (*F*), at *m/z* 544.1254 (2–) (*G*), and at *m/z* 572.6363 (2–) (*G*), all at NCE 70%, corresponding to tetrasaccharide linkage regions carrying one Neu5Ac residue and one sulfate group with Ser-Ala, Ser, and Gly-Ser residues, respectively.

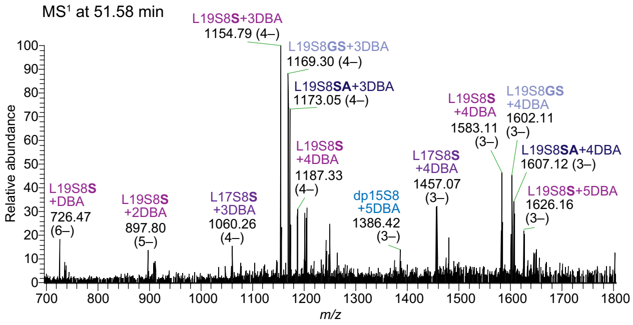

Figure S13. Intact CS glycopeptides from INS-1 832/13 cells identified after chondroitinase B depolymerization. MS[1] spectrum at 51.58 min displays variants of intact CS L19S8 precursor ions and charge state distribution after chondroitinase B depolymerization. The only difference from the spectrum shown in Fig. 5*A* is the presence of the precursor ion at *m/z* 1386.42 (3–) corresponding to the dp15S8 NRE oligosaccharide. The spectrum is displayed at the peak maximum of the precursor ion at *m/z* 1154.29 (4–).

References

1 Vizcaíno, J. A. *et al.* 2016 update of the PRIDE database and its related tools. *Nucleic Acids Res.* **44**, D447-D456, doi:10.1093/nar/gkv1145 (2016).

2 Ni, J., Pomerantz, S. C., Rozenski, J., Zhang, Y. & McCloskey, J. A. Interpretation of Oligonucleotide Mass Spectra for Determination of Sequence Using Electrospray Ionization and Tandem Mass Spectrometry. *Anal Chem.* **68**, 1989-1999, doi:10.1021/ac960270t (1996).

3 Linhardt, R. J., Avci, F. Y., Toida, T., Kim, Y. S. & Cygler, M. in *Adv Pharmacol* **53**, 187-215 (2006).

4 Murata, K., Ochiai, Y. & Akashio, K. Polydispersity of acidic glycosaminoglycan components in human liver and the changes at different stages in liver cirrhosis. *Gastroenterology* **89**, 1248-1257, doi:https://doi.org/10.1016/0016-5085(85)90640-7 (1985).

5 Persson, A., Vorontsov, E., Larson, G. & Nilsson, J. Glycosaminoglycan Domain Mapping of Cellular Chondroitin/Dermatan Sulfates. *Sci Rep.* **10**, 3506, doi:10.1038/s41598-020-60526-0 (2020).
