# Supplementary material for: Domain Mapping of Chondroitin/Dermatan Sulfate Glycosaminoglycans Enables Structural Characterization of Proteoglycans
Source: Mol Cell Proteomics. 2021 Mar 20;20:100074. doi: 10.1016/j.mcpro.2021.100074 (PMC8724862; doi:10.1016/j.mcpro.2021.100074)
Supplement: Supplemental Table S1 [file mmc2.pdf]

Table S1. Experimental and theoretical masses, and accuracy of precursor ions.

Structure	Composition	Experimental mass*	Theoretical mass	z	Acc. (ppm)	Figure reference	Reference file #MS^2 scan no.
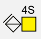	Δdp2S1	458.0598<br>458.0599	458.0610	1	-2.6<br>-2.4	2B<br>S10C	200626_08 #2628<br>200626_16 #2692
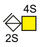	Δdp2S2	268.5056	268.5053	2	1.3	2C	200214_14 #6633
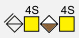	Δdp4S2	458.0608	458.0610	2	-0.4	S10D	200626_16 #5699
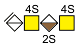	Δdp4S3	562.6158	562.6153	2	0.9	3D	200214_17 #10278
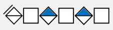	Δdp6S0	567.6596<br>567.6606<br>567.6612	567.6600	2	-0.6<br>1.1<br>2.2	S6A<br>S6C<br>S6E	200603_28 #3202<br>200603_94 #3269<br>200214_14 #5992
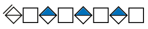	Δdp8S0	757.2163<br>757.2158<br>757.2169	757.2157	2	0.8<br>0.1<br>1.6	S6B<br>S6D<br>S6F	200603_28 #3906<br>200603_94 #4533<br>200214_14 #7762
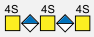	dp5S3	673.1607	673.1603	2	0.6	S10E	200214_20 #9471
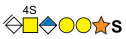	ΔL6S1Ser	588.1340	588.1329	2	2.0	4B<br>S11D	200130_36 #5639
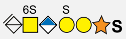	ΔL6S2Ser	628.1121	628.1113	2	1.4	4C<br>S11G	200130_36 #7844
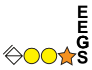	ΔL4S0Ser-Gly-Glu-Glu (EEGS)	516.1523	516.1521	2	0.5	4E<br>S12B	200214_17 #5234
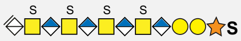	ΔL12S4Ser	850.8217	850.8211	3	0.7	4F	200626_24 #10626
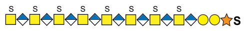	L19S8Ser	1154.2891	1154.2908	4	-1.5	5B	200626_31 #13267
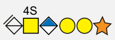	ΔL6S1	544.6180	544.6169	2	2.1	S11B	200214_04 #5747
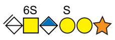	ΔL6S2	584.5966	584.5953	2	2.3	S11C	200214_14 #8053
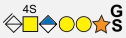	ΔL6S1Ser-Gly (GS)	616.6448	616.6436	2	1.9	S11E	200130_36 #5578
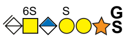	ΔL6S2Ser-Gly (GS)	623.6524	623.6514	2	1.6	S11F	200130_36 #5773
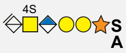	ΔL6S1Ser-Ala	656.6230	656.6220	2	1.5	S11H	200130_36 #7684
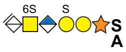	ΔL6S2Ser-Ala	663.6310	663.6298	2	1.8	S11I	200130_36 #7940
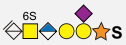	ΔL6S1SA1Ser	733.6813	733.6806	2	1.0	S11J	200214_14 #7711
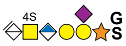	ΔL6S1SA1Ser-Gly (GS)	762.1917	762.1913	2	0.5	S11K	200214_14 #7558
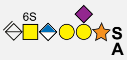	ΔL6S1SA1Ser-Ala	769.1995	769.1991	2	0.5	S11L	200214_14 #7801
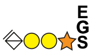	ΔL4S0Ser-Gly-Glu (EGS)	451.6309	451.6308	2	0.3	S12A	200214_17 #3906
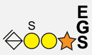	ΔL4S1Ser-Gly-Glu (EGS)	491.6093	491.6092	2	0.3	S12C	200214_17 #5586
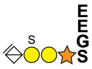	ΔL4S1Ser-Gly-Glu-Glu (EEGS)	556.1306	556.1305	2	0.3	S12D	200214_17 #6931
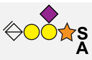	ΔL4S0SA1Ser-Ala	539.6652	539.6650	2	0.5	S12E	200214_17 #4166

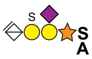	ΔL4S1SA1Ser-Ala	579.6435	579.6434	2	0.3	S12F	200214_17 #5907
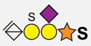	ΔL4S1SA1Ser	544.1254	544.1248	2	1.1	S12G	200214_17 #5833
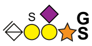	ΔL4S1SA1Ser-Gly (GS)	572.6363	572.6356	2	1.3	S12H	200214_17 #5731
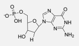	dGMP	346.0566	346.0558	1	2.3	S3A	200214_14 #4035
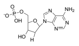	dAMP	330.0612	330.0609	1	0.9	S3B	200214_14 #4399
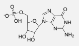	GMP	362.0511	362.0507	1	1.1	S3C	200130_36 #4032
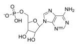	AMP	346.0565	346.0558	1	2.0	S3D	200130_36 #4399
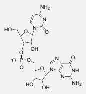	CG	587.1266	587.1257	1	1.5	S3E	200130_36 #4016
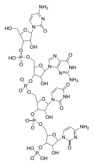	CGUC	638.5772	638.5757	2	2.4	S3F	200130_36 #8595

*Depending on the spectrum scan number, the two last decimals differ slightly. This explains any differences between the presented decimal values in the tables and figures. Experimental monoisotopic masses were taken directly prior to the reference MS^2 scan number
