# Supplementary material for: Domain Mapping of Chondroitin/Dermatan Sulfate Glycosaminoglycans Enables Structural Characterization of Proteoglycans
Source: Mol Cell Proteomics. 2021 Mar 20;20:100074. doi: 10.1016/j.mcpro.2021.100074 (PMC8724862; doi:10.1016/j.mcpro.2021.100074)
Supplement: Supplemental Table S2 [file mmc3.pdf]

Table S2. Annotations, experimental and theoretical masses, and accuracies of fragment ions included in Fig. 2, 4, 5, S6, S11, and S12. Fragment ions previously annotated by us are not included[1].

Proposed structure	Experimental mass	Theoretical mass	*z*	Accuracy (ppm)	Figure reference
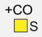	328.0345	328.0344	1	-0.3	2C
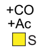	370.0446	370.0450	1	1.1	2C
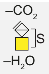	396.0625	396.0606	1	-4.8	2C
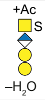	411.5855	411.58495	2	-1.3	4B
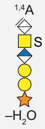	505.6012	505.60095	2	-0.5	4B
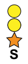	560.1845	560.1832	1	-2.3	4B
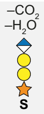	674.2158	674.2149	1	-1.3	4B
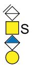	796.1461	796.1459	1	-0.3	4B
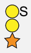	553.1088	553.1080	1	-1.4	4C
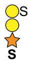	640.1407	640.1400	1	-1.1	4C
**EGS** –CO_2 –$2H_2O$	210.0887	210.0883	1	-1.9	4E
**EEGS** –CO_2 –$2H_2O$	339.1313	339.1309	1	-1.2	4E
**EEGS** –CO_2 –H_2O	357.142	357.1415	1	-1.4	4E
**EEGS** –H_2O	401.1318	401.1313	1	-1.2	4E
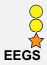	437.1379	437.14125	2	7.7	4E
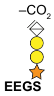	494.1574	494.1571	2	-0.6	4E
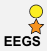	713.2371	713.2370	1	-0.1	4E
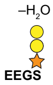	857.2803	857.2792	1	-1.3	4E
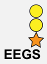	875.2907	875.2898	1	-1.0	4E
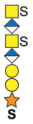	738.6541	738.65625	2	2.9	4F

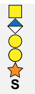	939.2938	939.2947	1	1.0	4F
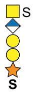	1019.2515	1019.2515	1	0.0	4F
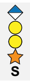	736.2136	736.2153	1	2.3	5B
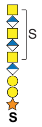	888.233	888.2336	2	0.7	5B
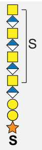	1077.7791	1077.78935	2	9.5	5B
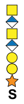	1318.4004	1318.4062	1	4.4	5B
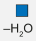	202.0725	202.0721	1	-2.0	S5A
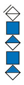	466.1205<br>933.2491	466.12025<br>933.2478	2<br>1	-0.5<br>-1.4	S5A
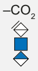	510.1471	510.1465	1	-1.2	S5A
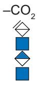	713.2265	713.2259	1	-0.8	S5A
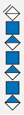	655.6766	655.676	2	-0.9	S5B
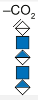	889.2585	889.2580	1	-0.6	S5B
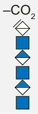	1092.3384	1092.3374	1	-0.9	S5B
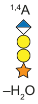	571.1521	571.1515	1	-1.1	S10B
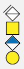	716.1896	716.1891	1	-0.7	S10B
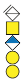	878.2438	878.2419	1	-2.2	S10B
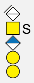	958.1997	958.1987	1	-1.0	S10B
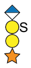	729.1426	729.1401	1	-3.4	S10C
GS $-H_2O$	143.0465	143.0461	1	-2.8	S10E
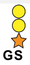	617.2097	617.2046	1	-8.3	S10E

SA $-H_2O$	157.0622	157.0618	1	-2.5	S10F
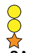 SA	631.2216	631.2203	1	-2.1	S10F
$-CO_2$ $-H_2O$ 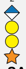 SA	745.2521	745.2520	1	-0.1	S10H
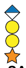 SA	807.2527	807.2524	1	-0.4	S10I
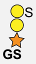 GS	697.1615	697.1614	1	-0.1	S10J
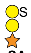 SA	711.1774<br>355.0851	711.1771<br>355.0849	1<br>2	-0.4<br>-0.6	S10J<br>S11F
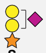 S	851.2794	851.2786	1	-0.9	S10J
$-CO_2$ $-H_2O$ 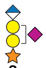 S	965.3118	965.3103	1	-1.6	S10J
$-CO_2$ $-H_2O$ 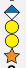 S	731.2375	731.2363	1	-1.6	S10K
$-CO_2$ $-H_2O$ 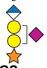 GS	1022.3348	1022.3317	1	-3.0	S10K
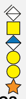 GS	1154.3395	1154.3376	1	-1.6	S10K
$-H_2O$ 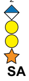 SA	789.2427	789.2418	1	-1.1	S10L
$-CO_2$ 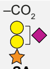 SA	878.3282	878.3259	1	-2.6	S10L
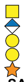 SA	1010.3214	1010.3318	1	10.3	S10L
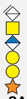 SA	1168.3544	1168.3533	1	-0.9	S10L
EGS $-CO_2-H_2O$	228.0996	228.0989	1	-3.1	S11A
EGS $-H_2O$	272.089	272.0887	1	-1.1	S11A
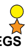 EGS	291.5931<br>584.1943	291.5935<br>584.1944	2<br>1	1.5<br>0.2	S11A
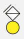	337.0776	337.0776	1	0.0	S11A
$-H_2O$ 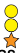 EGS	728.2357	728.2366	1	1.2	S11A

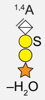	316.0456	316.0452	2	-1.3	S11C
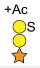	595.119	595.1186	1	-0.7	S11C
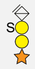	711.1296	711.1295	1	-0.1	S11C
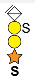	798.1581	798.1615	1	4.3	S11C
E–H_2O	128.0356	128.0353	1	-2.3	S11D
EG –H_2O	185.0569	185.0567	1	-1.1	S11D
EEG –H_2O	314.0997	314.0993	1	-1.3	S11D
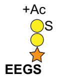	498.1252	498.12495	2	-0.5	S11D
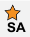	307.1147	307.1147	1	0.0	S11E
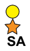	469.1678	469.1675	1	-0.6	S11E
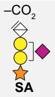	517.6699	517.67005	2	0.3	S11E
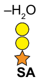	613.2095	613.2097	1	0.3	S11E
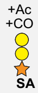	701.2256	701.2258	1	0.3	S11E
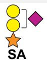	922.3057	922.3157	1	10.8	S11E
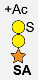	376.0903	376.0902	2	-0.3	S11F
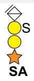	434.0957	434.09565	2	-0.1	S11F
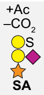	499.643	499.643	2	0.0	S11F
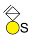	208.0138<br>417.0347	208.01355<br>417.0344	2<br>1	-1.2	S11G
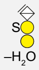	280.0349	280.03465	2	-0.9<br>-0.7	S11G
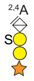	310.0456	310.04525	2	-1.1	S11G
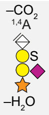	439.5982	439.598	2	-0.5	S11G
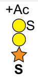	682.1507	682.1506	1	-0.1	S11G
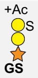	739.1721	739.1720	1	-0.1	S11H
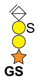	855.1846	855.1829	1	-2.0	S11H

Reference

1 Persson, A., Vorontsov, E., Larson, G. & Nilsson, J. Glycosaminoglycan Domain Mapping of Cellular Chondroitin/Dermatan Sulfates. *Sci Rep.* **10**, 3506, doi:10.1038/s41598-020-60526-0 (2020).
